# Levels of Ycg1 Limit Condensin Function during the Cell Cycle

**DOI:** 10.1371/journal.pgen.1006216

**Published:** 2016-07-27

**Authors:** Tyler W. Doughty, Heather E. Arsenault, Jennifer A. Benanti

**Affiliations:** Department of Molecular, Cell and Cancer Biology, University of Massachusetts Medical School, Worcester, Massachusetts, United States of America; Université de Montréal, CANADA

## Abstract

During mitosis chromosomes are condensed to facilitate their segregation, through a process mediated by the condensin complex. Although several factors that promote maximal condensin activity during mitosis have been identified, the mechanisms that downregulate condensin activity during interphase are largely unknown. Here, we demonstrate that Ycg1, the Cap-G subunit of budding yeast condensin, is cell cycle-regulated with levels peaking in mitosis and decreasing as cells enter G1 phase. This cyclical expression pattern is established by a combination of cell cycle-regulated transcription and constitutive degradation. Interestingly, overexpression of *YCG1* and mutations that stabilize Ycg1 each result in delayed cell-cycle entry and an overall proliferation defect. Overexpression of no other condensin subunit impacts the cell cycle, suggesting that Ycg1 is limiting for condensin complex formation. Consistent with this possibility, we find that levels of intact condensin complex are reduced in G1 phase compared to mitosis, and that increased Ycg1 expression leads to increases in both levels of condensin complex and binding to chromatin in G1. Together, these results demonstrate that Ycg1 levels limit condensin function in interphase cells, and suggest that the association of condensin with chromosomes must be reduced following mitosis to enable efficient progression through the cell cycle.

## Author Summary

Chromosome conformation is cell cycle-regulated so that chromosomes are highly compacted to facilitate their segregation during mitosis, and decondensed during interphase to facilitate DNA-dependent processes such as replication and transcription. Understanding how chromosomes transition between these different states is important for understanding how cells maintain a stable genome. The condensin complex is an essential five-subunit complex that controls chromosome condensation in all eukaryotes. In this study, we show that expression of the Cap-G/Ycg1 subunit of condensin in budding yeast is cell cycle-regulated, and that its reduced expression during interphase limits condensin function. When this regulation is disrupted, and Ycg1 is constitutively expressed, progression through interphase is delayed. Emerging evidence indicates that individual condensin subunits are also expressed at limiting levels in metazoan cells, which suggests that cell-cycle regulation of an individual condensin subunit is a conserved mechanism that coordinates condensin function with the cell cycle.

## Introduction

The eukaryotic cell cycle is divided into two distinct parts: interphase, when cell growth and DNA replication occur, and mitosis, when chromosomes are segregated into daughter cells. One major phenotypic difference between these phases is chromosome conformation. Specifically, interphase chromosomes are decondensed and loosely packed within the nucleus, which allows for maximum accessibility of the DNA to the transcription and replication machineries, while mitotic chromosomes are tightly compacted and condensed, which facilitates their segregation during anaphase [1]. Accurate transit in and out of these conformations is paramount to proliferation, since decondensed chromosomes during mitosis impede segregation, and can generate DNA breaks that lead to genome instability [2,3], whereas condensed chromosomes during interphase hinder transcription and replication, and thus may impede cell-cycle progression.

One important factor involved in controlling interphase and mitotic chromosome conformations is the condensin complex [4]. Condensin is a conserved eukaryotic complex that is comprised of five protein subunits: two core ATPase subunits (Smc2 and Smc4), a kleisin subunit (CAP-H/Brn1), and two HEAT-repeat subunits (CAP-G/Ycg1 and CAP-D2/Ycs4), each of which is essential for complex function and cell viability [5–8]. Mammalian cells have two condensin complexes, condensin I and condensin II, which differ in their non-SMC subunits and mediate different aspects of chromosome condensation [9,10]. In contrast, yeast have only one complex, which is similar in sequence to condensin I in mammals [11].

In all organisms, condensin function is most pronounced during mitosis, when its phosphorylation-stimulated activity leads to large-scale supercoiling of DNA and chromosome compaction [12,13]. After the completion of mitosis, condensin supercoiling activity decreases, resulting in chromosome decondensation [13,14]. Although supercoiling activity is diminished after mitosis, some condensin remains associated with chromatin throughout interphase. In budding yeast, condensin associates with genes encoding tRNAs, ribosomal proteins, and small nuclear and nucleolar RNAs (*SNR* genes) throughout the cell cycle and aids in clustering of these loci [15–17]. Condensin also has non-mitotic roles in establishing metazoan chromosome structure [18–21]. However, the mechanisms that coordinate these different condensin functions with the appropriate cell-cycle stage are not well understood.

Previous studies investigating condensin regulation have mainly focused on how phosphorylation activates the complex during mitosis to trigger chromosome condensation. Condensin phosphorylation by Polo kinase, Aurora B, and Cdk1 has been shown to promote its localization to mitosis-specific loci, and to stimulate its supercoiling activity [13,14,22–24]. In addition, binding of budding yeast condensin to centromeres and the repetitive rDNA locus increases during mitosis via recruitment by Sgo1 and Fob1, respectively, which act as chromatin-associated receptors [25–27]. Much less is known about how chromosome condensation is reversed after mitosis is complete. However, changes in condensin phosphorylation upon mitotic exit are likely to play a role in this process. Specifically, mitotic kinases are inactivated in late mitosis [28], and inhibitory phosphorylation by CKII may limit condensin activity in interphase, as has been demonstrated for human condensin [29]. In mammals, condensin I relocalizes to the cytoplasm in interphase [30,31], thereby restricting its access to chromosomes. However, mammalian condensin II and budding yeast condensin are constitutively nuclear [5,30,31], and thus are predicted to have additional mechanisms to regulate their association with chromosomes. The precise mechanisms that downregulate the activity of these complexes after mitosis are not known.

Emerging evidence suggests that proteasomal degradation of an individual subunit may be one mechanism that limits condensin activity. In *Drosophila melanogaster*, the Cap-H2 subunit of condensin II is targeted for ubiquitin-mediated degradation, and blocking this degradation results in partial chromosome condensation in interphase cells [32,33]. Additional studies have reported ubiquitination of the Cap-G subunit in budding yeast [34], and that human condensin II subunits are degraded by the ubiquitin-proteasome system [35,36]. However, it is not known in any system if ubiquitin-mediated degradation leads to cyclical expression of any condensin subunit during the cell cycle, and if levels of a subunit do cycle, whether or not this regulation contributes to cell cycle-regulated changes in chromosome structure.

In this report, we show that the Cap-G subunit of budding yeast condensin (Ycg1) is expressed in a cell cycle-dependent manner due to cyclical transcription coupled with constitutive degradation. Further, we observe that cyclical expression maintains Ycg1 at limiting levels relative to the other condensin subunits. Finally, we show that increasing Ycg1 expression results in increased recruitment of condensin complex to chromosomes during G1 phase, and interferes with progression through the G1/S transition. These data suggest that downregulation of Ycg1 after mitosis contributes to a reduction in condensin activity, and that a decrease in condensin function during G1 phase is necessary to facilitate cell-cycle progression.

## Results

### Ycg1 protein and transcript levels are cell cycle-regulated

**Fig 1:**
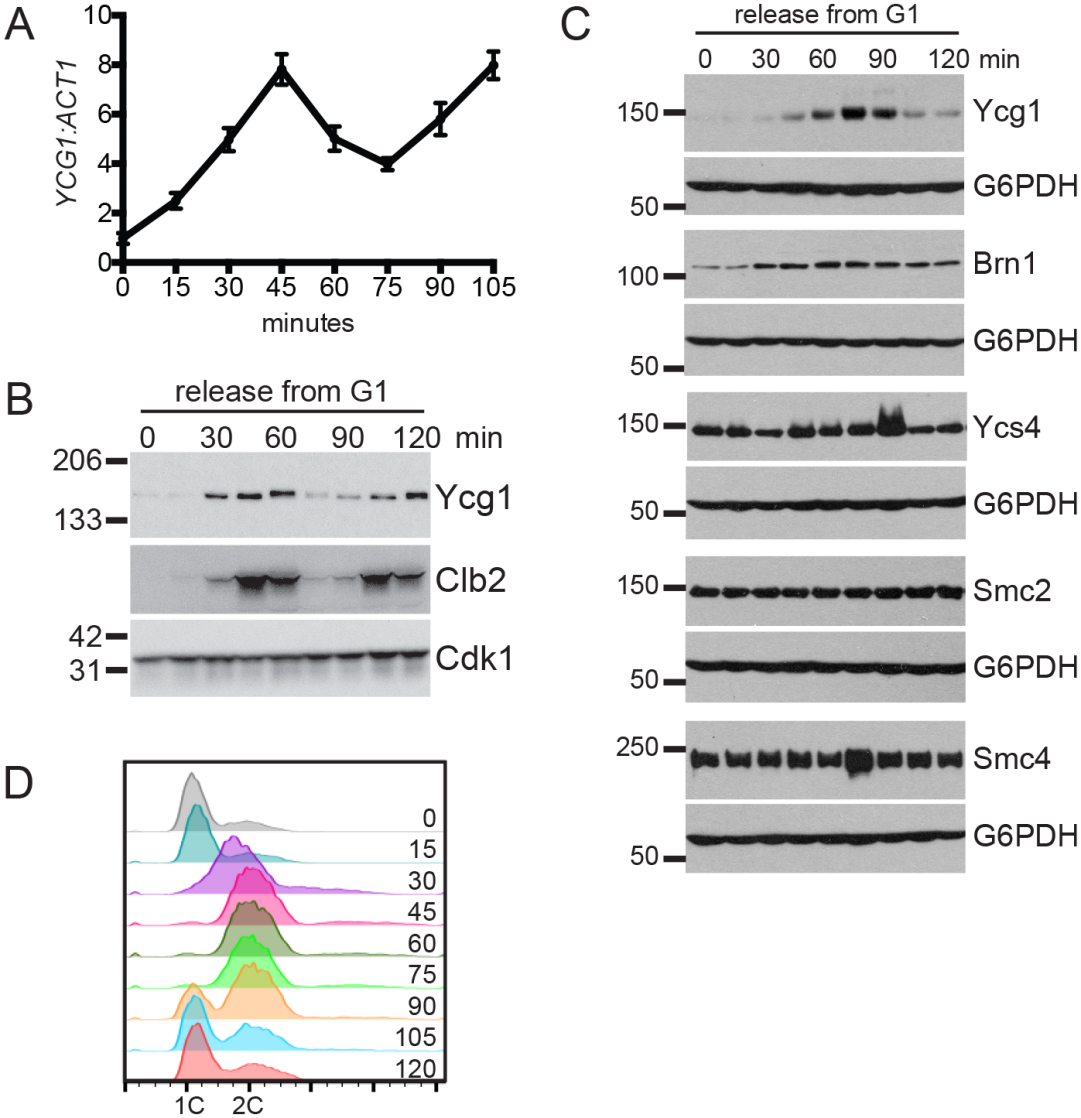
Ycg1 expression is cell cycle-regulated. **(A)** *YCG1* mRNA levels following release from G1 arrest. Wild-type cells (YBL320) were arrested in G1 with alpha-factor for 2 hours, released into the cell cycle, and gene expression changes were analyzed by RT-qPCR. *YCG1* levels are shown relative to *ACT1*. Time course samples were previously analyzed by DNA microarray in [40]. (**B)** Wild-type cells expressing Ycg1-GFP (YCG1-GFP) were arrested in G1 with alpha-factor for 3 hours then released into fresh medium, as in (A). Western blot samples were collected every 15 minutes. Clb2 is shown as a marker of mitosis and Cdk1 is shown as a loading control. **(C)** Wild-type strains expressing the individual condensin subunits tagged with a 3HA tag (YTD33, YTD82, YTD83, YTD84, YTD80) were arrested in G1 with alpha-factor for 3 hours, released into the cell cycle, and samples taken for Western blot and flow cytometry every 15 minutes. Alpha-factor was added back after 45 minutes to arrest cells in the subsequent G1 phase. **(D)** Representative plot showing DNA content following release from G1 arrest in (C), data is from the YTD33 time course. All other strains showed nearly identical plots. Note that the strains in used in parts A and B are in a different strain background than those used in parts C and D (S288C compared to W303), and the timing of cell cycle-progression differs slightly.

Although the budding yeast condensin complex associates with chromatin throughout the cell cycle [6,7,15,17], its activity increases substantially during mitosis. Previous reports have shown that this change in activity is due in part to increased phosphorylation [3,14,24], and to enhanced recruitment of the complex to a subset of sites in the genome [15,17,25–27,37]. Interestingly, several studies have also reported that transcription of the gene encoding the Cap-G subunit of condensin, *YCG1*, is cell cycle-regulated (Fig 1A) [38–40], with lower levels in G1 than mitosis. Additionally, Ycg1 protein levels have been reported to be lower in interphase than in mitosis [22]. This evidence suggests that regulation of Ycg1 levels may be an additional mechanism that coordinates condensin activity with the cell cycle. To investigate this possibility further, we examined expression of Ycg1 mRNA and protein following release from a G1 arrest and found that they cycled similarly: expression increased as cells progressed through interphase, peaked during mitosis, and declined upon entry into the next G1 phase, similar to the mitotic cyclin Clb2 (Fig 1A and 1B). In contrast, none of the other subunits of the condensin complex displayed this dramatic fluctuation during the cell cycle, although Brn1 expression was modestly decreased in G1-arrested cells (Fig 1C and 1D). These observations, coupled with the fact that Ycg1 is essential for condensin function [7,17,22], suggest that regulation of Ycg1 levels during the cell cycle may be a previously uncharacterized mechanism that limits condensin function during interphase.

### Ycg1 undergoes proteasomal degradation throughout the cell cycle

**Fig 2:**
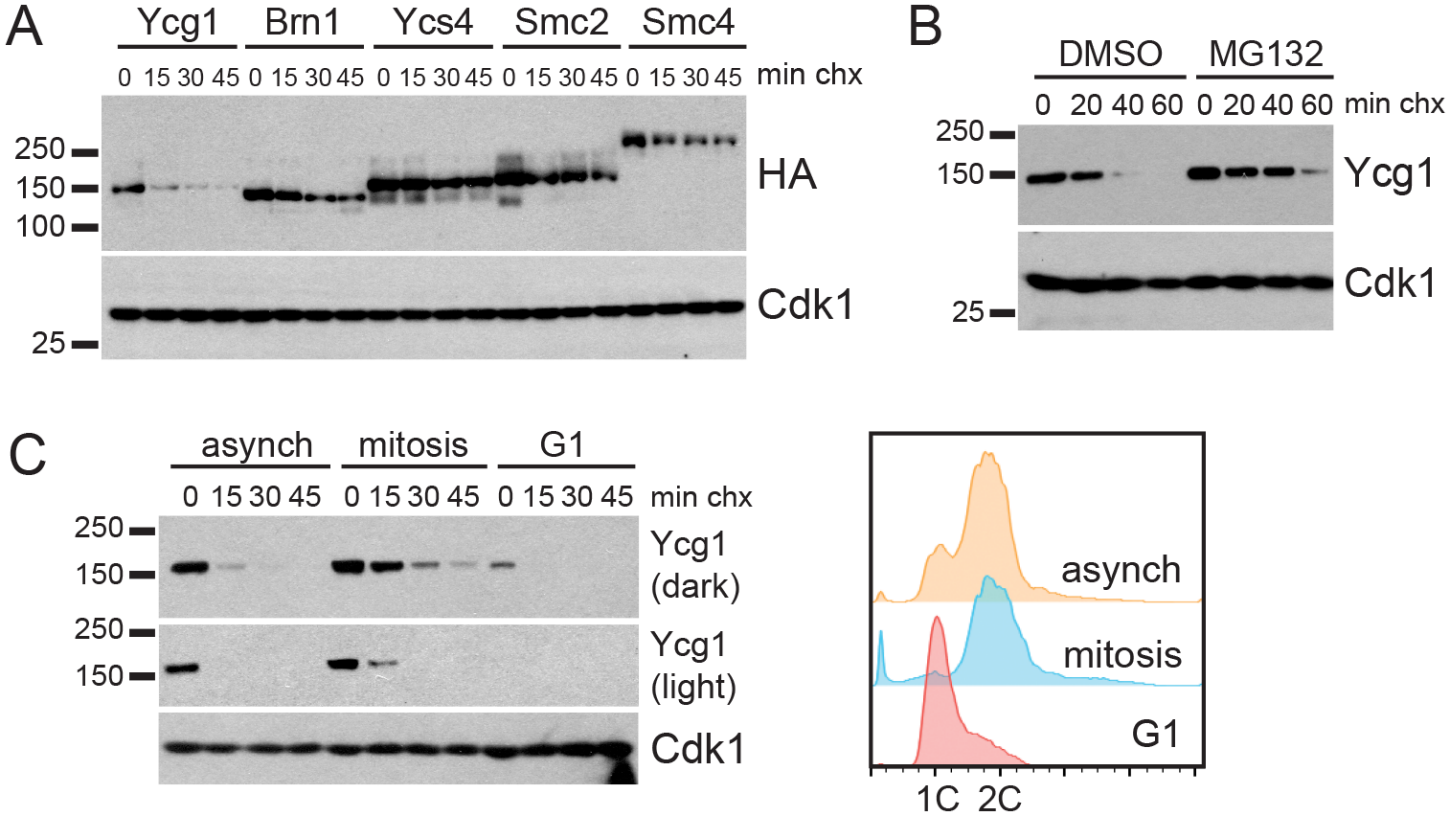
Ycg1 undergoes proteasomal degradation throughout the cell cycle. **(A)** Asynchronous cells harboring the indicated 3HA-tagged condensin subunits (YTD33, YTD82, YTD83, YTD84, YTD80) were treated with cycloheximide (chx) and samples were collected every 15 minutes. HA and Cdk1 Western blots are shown. **(B)** Asynchronous cells expressing Ycg1-3HA (YTD43) were incubated for 2 hours with DMSO (control) or the proteasome inhibitor MG132, then a cycloheximide-chase assay was performed, as in (A). **(C)** Cells expressing Ycg1-3HA (YTD33) were arrested with nocodazole (mitosis), alpha-factor (G1), or left untreated (asynch), and cycloheximide-chase assays were performed. Shown are Western blots examining Ycg1 (dark and light exposures of the same blot) and Cdk1 (left), as well as flow cytometry plots to confirm cell-cycle distributions (right).

The rapid decrease in Ycg1 levels after mitosis suggested that Ycg1 might also be regulated by proteolysis. To test this possibility and assay its stability, we monitored Ycg1 levels in asynchronous cells over time in the presence of the translation inhibitor cycloheximide, and found that Ycg1 was rapidly degraded (Fig 2A). Next, we asked whether other subunits of the complex were similarly regulated. To do this, each subunit of the complex was tagged with an identical 3HA tag, and their stabilities were compared in the same assay. This analysis revealed that Ycg1 is the least stable, and the least abundant, subunit of the condensin complex (Fig 2A).

Many cyclically expressed proteins are degraded by the ubiquitin proteasome system (UPS) [41], and Ycg1-ubiquitin conjugates were previously identified in a proteomic screen [34], which suggested that Ycg1 may undergo ubiquitin-mediated degradation. Consistent with this possibility, proteasomal inhibition impaired Ycg1 turnover in asynchronous cells, confirming that the protein is regulated by the UPS (Fig 2B). Since Ycg1 is necessary for condensin function, and condensin function is essential for the completion of mitosis [7,17,22], we speculated that Ycg1 might be stable during mitosis. To test this, we arrested cells in G1 or mitosis, and monitored Ycg1 turnover (Fig 2C). We found that although there was more protein in mitosis, consistent with its increased transcription late in the cell cycle (Fig 1A), Ycg1 was degraded in both arrests. This observation suggests that Ycg1 is degraded throughout the cell cycle, surprisingly, even during mitosis. Taken together, these data indicate that constitutive degradation, paired with cyclical transcription, leads to cell cycle-regulated expression of Ycg1.

### The C-terminus of Ycg1 is necessary for its degradation

**Fig 3:**
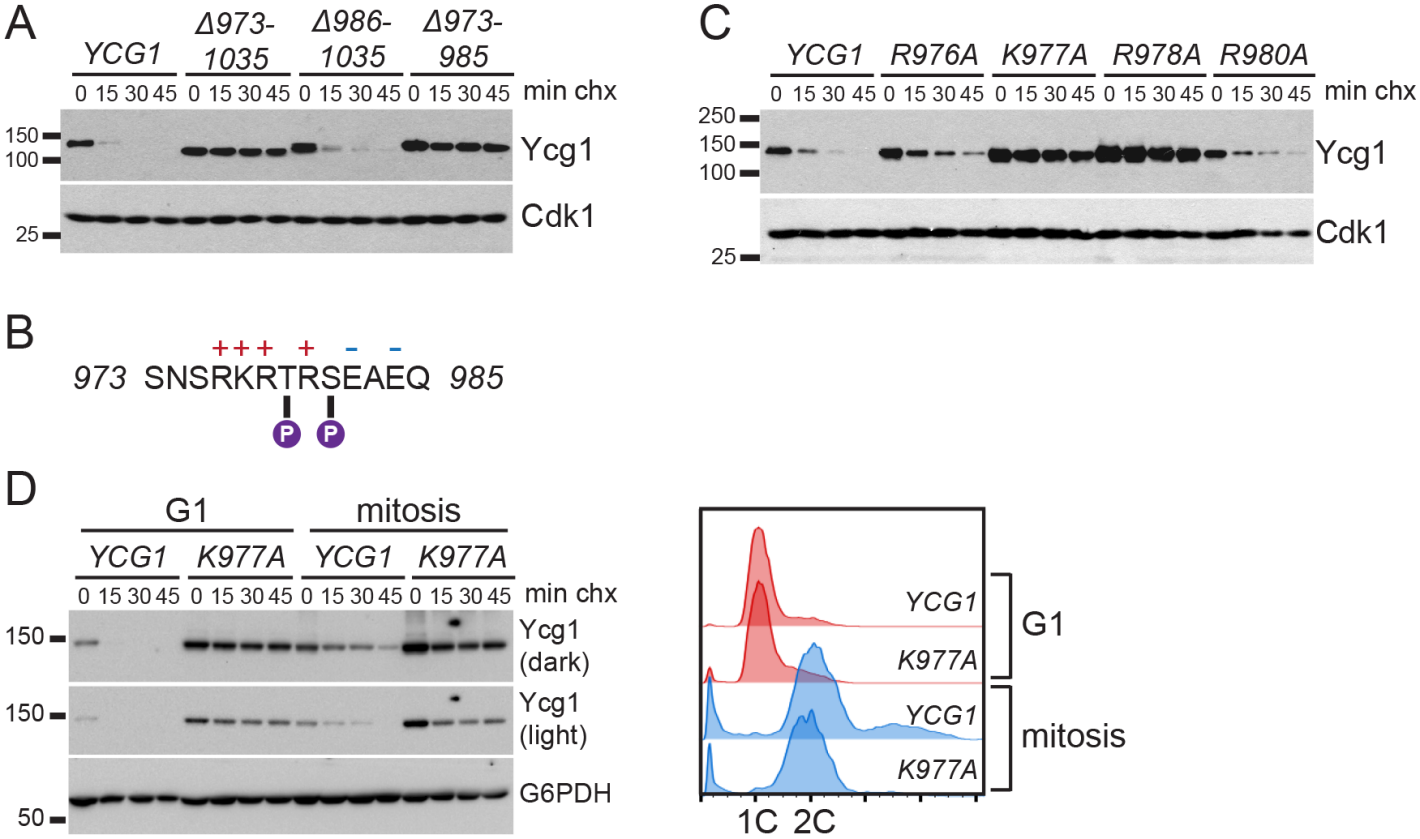
The C-terminus of Ycg1 regulates its degradation. **(A)** Cycloheximide-chase assay showing turnover of 3HA-tagged wild-type Ycg1 (YTD33) and the indicated deletion mutants (YTD36, YTD184, YTD128) in asynchronous cells. Also see S1A Fig for an illustration of the mutants. Western blots for HA and Cdk1 (loading control) are shown. **(B)** Sequence of Ycg1 amino acids 973–985 that regulate Ycg1 stability. Charged amino acids are indicated, as well as T979 and S981 which have been previously shown to be phosphorylated [14]. **(C)** Cycloheximide-chase assay of strains expressing the indicated 3HA-tagged Ycg1 proteins (YTD33, YTD200, YTD148, YTD201, YTD164) in asynchronous cells. Western blots for HA and Cdk1 (loading control) are shown. **(D)** *YCG1* (YTD33) and *ycg1-K977A* (YTD148) strains were arrested in G1 with alpha-factor or in mitosis with nocodazole for 3 hours and cycloheximide-chase assays performed. Western blots for HA and G6PDH (loading control) are shown. Flow cytometry plots (right) confirm cell-cycle arrests.

Next, we sought to investigate the importance of cyclical Ycg1 expression for progression through the cell cycle. To do this, we engineered mutations in Ycg1 that blocked degradation. Most proteins that undergo ubiquitin-mediated degradation have short sequences termed degrons, which are essential for degradation. Many degron sequences are found in unstructured domains that are subject to other forms of regulation, such as phosphorylation [42]. Interestingly, the C-terminal domain of Ycg1 fits these criteria [14,43]. Moreover, although this domain includes several phosphorylation sites that contribute to condensin activation during mitosis, this domain is not essential for viability [14], which allowed us to replace the endogenous copy of *YCG1* with alleles carrying mutations in this region. We first tested whether this domain was required for Ycg1 degradation and found that Ycg1 was completely stabilized when the C-terminal 63 amino acids were deleted (Fig 3A, Ycg1Δ973–1035). However, deletion of the C-terminal 50 amino acids had no effect on Ycg1 degradation (Ycg1Δ986–1035). These data suggested that Ycg1 turnover requires amino acids 973–985 and, consistent with this possibility, deletion of these amino acids was sufficient to stabilize the protein (Ycg1Δ973–985, Fig 3A). Additional deletions and truncations in the C-terminus were consistent with this conclusion (S1A Fig).

Since amino acids 973–985 lie within the conserved phosphoregulatory domain of Ycg1 (S1A Fig) [43], we endeavored to create a stable mutant that minimally alters the sequence of this region. To do this we mutated features within this region that might contribute to degradation, including charged residues and putative phosphorylation sites (Fig 3B). We found that positively charged residues were necessary for Ycg1 degradation, with mutation of lysine-977 or arginine-978 having the greatest effect (Fig 3C). In contrast, mutation of negatively charged residues, or all serines and threonines in the region, had little to no effect on Ycg1 stability (S1B Fig). Although our data suggest that Ycg1 is degraded throughout the cell cycle (Fig 2C), we confirmed that the increased stability of Ycg1-K977A did not result from a change in cell-cycle distribution in the mutant strain by arresting cells in G1 or mitosis and assaying Ycg1 turnover. This analysis confirmed that Ycg1-K977A is more stable than wild-type Ycg1 in both phases of the cell cycle (Fig 3D).

### Constitutive Ycg1 expression delays cell-cycle entry

**Fig 4:**
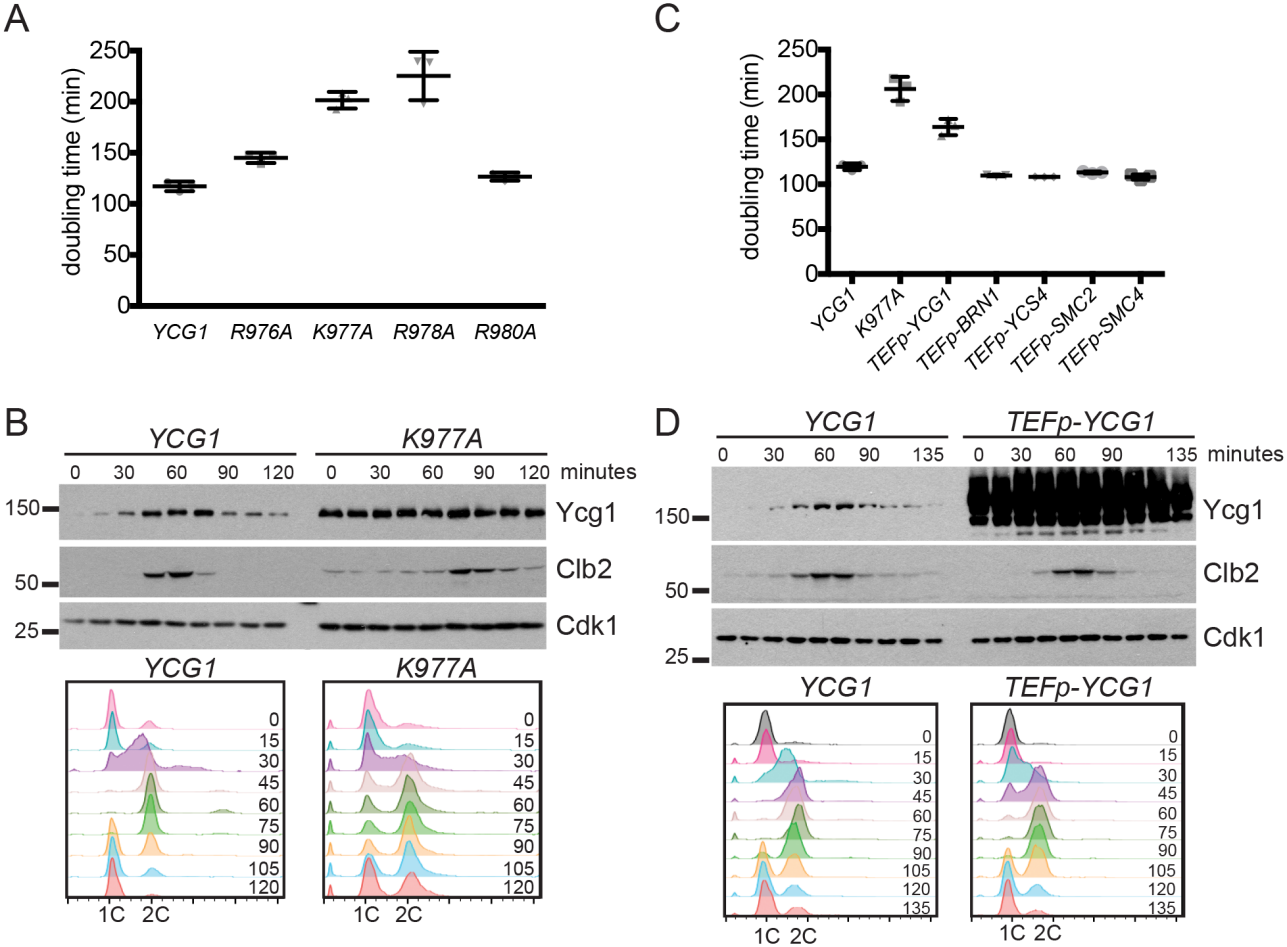
Constitutive expression of Ycg1 delays progression through the cell cycle. **(A)** Doubling time of strains expressing the indicated Ycg1 proteins from the endogenous locus (YTD33, YTD200, YTD148, YTD201, YTD164). Mean doubling time from 3 independent experiments, +/- 1 standard deviation, are shown. **(B)** Strains expressing 3HA-tagged Ycg1 (YTD33) or Ycg1-K977A (YTD148) from the endogenous locus were arrested in G1 with alpha-factor for 3 hours and then released into the cell cycle. Alpha-factor was added back after 45 minutes to prevent cells from entering a second cycle. Western blots of Ycg1-3HA, Clb2 and Cdk1 are shown (top). Flow cytometry plots (bottom) illustrate the delayed progression of *ycg1-K977A* cells into S phase (compare 30 and 45-minute time points). **(C)** Doubling time of wild-type (YTD33) and *ycg1-K977A* strains (YTD148) were compared to strains overexpressing each of the indicated condensin subunits from the *TEF1* promoter (YTD336, YTD337, YTD353, YTD349, YTD362). Mean doubling times from 3 independent experiments are shown, +/- 1 standard deviation. **(D)** *YCG1* (YTD276) and *TEFp-YCG1* (YTD361) strains were arrested in metaphase with a *MET3p-CDC20* shut-off allele for 3 hours, then released into alpha-factor for 2 hours to synchronize cells in G1. Alpha-factor was added back 45 minutes after release from G1 arrest to prevent cells from entering a second cycle. Western blots of Ycg1-3HA, Clb2 and Cdk1 are shown (top). Flow cytometry plots (bottom) demonstrate the delayed progression of *ycg1-K977A* cells into S phase (compare 30 and 45-minute time points).

The prevailing model suggests that chromosome condensation needs to be reversed after mitosis to facilitate essential DNA-dependent processes during interphase, such as replication and transcription. Since Ycg1 is downregulated after mitosis, we posited that interference with this regulation might impact cell-cycle progression. To test this, we analyzed the proliferation rate of each of the strains expressing point mutations that stabilize Ycg1. Interestingly, we observed a modest increase in doubling time in mutants that partially blocked Ycg1 turnover, and a much larger increase in doubling time in mutants that fully blocked turnover (Fig 4A, S1B Fig). These data show a correlation between increased Ycg1 expression and decreased proliferation rate, suggesting that Ycg1 downregulation after mitosis may be important for cell-cycle progression.

Next, we asked whether the decreased proliferation rate that we observed in cells expressing stable Ycg1 resulted from a delay at a specific point in the cell cycle. Strains expressing Ycg1 or Ycg1-K977A were synchronized in G1 phase and released. Cell-cycle progression was then followed by flow cytometry and Ycg1 levels were monitored by Western blot. In contrast to the wild-type protein, Ycg1-K977A was expressed at a constant level throughout the cell cycle (Fig 4B, top), demonstrating that degradation is necessary for cell cycle-dependent changes in Ycg1 levels. Notably, *ycg1-K977A* strains exhibited delayed progression from G1 into S phase (Fig 4B, bottom), consistent with the possibility that failing to downregulate condensin might interfere with progression through interphase.

Haploid *ycg1-K977A* strains are viable, confirming that the allele encodes a functional protein. However, the K977A mutation falls in a domain of Ycg1 that is required for maximal condensin activity [14], raising the possibility that this mutation might both increase Ycg1 expression and reduce its function. To address this possibility, we performed additional characterization of *ycg1-K977A* strains. First, we confirmed that the interaction between Ycg1-K977A and the other subunits of condensin was not impaired (S2A Fig). In addition, we used an established rDNA reporter assay [44] to investigate whether *ycg1-K997A* cells exhibited defects in rDNA silencing, or increased recombination at the rDNA locus, both of which are phenotypes exhibited by condensin loss-of-function mutants [5,6]. We found that *ycg1-K977A* cells were similar to wild-type cells in this assay (S2B Fig). Moreover, the proliferation defect in *ycg1-K977A* strains could not be rescued by the addition of a second copy of *YCG1* integrated at the *URA3* locus, suggesting that the growth defect is not the result of reduced function of the mutant (S2F Fig). Although these assays suggested Ycg1-K977A is functional, we observed that multiple isolates of haploid *ycg1-K977A* strains exhibited non-uniform colony size (S2C Fig), exhibited increased sensitivity to the replication inhibitor hydroxyurea (HU) (S2D Fig) (a phenotype that has been reported for strains expressing hypomorphic alleles of condensin subunits in fission yeast [45]), and showed increased sensitivity to the microtubule poison benomyl (S2E Fig). Moreover, we had difficulty generating haploid strains that expressed Ycg1-K977A and had an epitope tag on any other subunit of the condensin complex. Together, these findings suggested that the K977A mutation might reduce Ycg1 function, in addition to stabilizing the protein.

**Fig 5:**
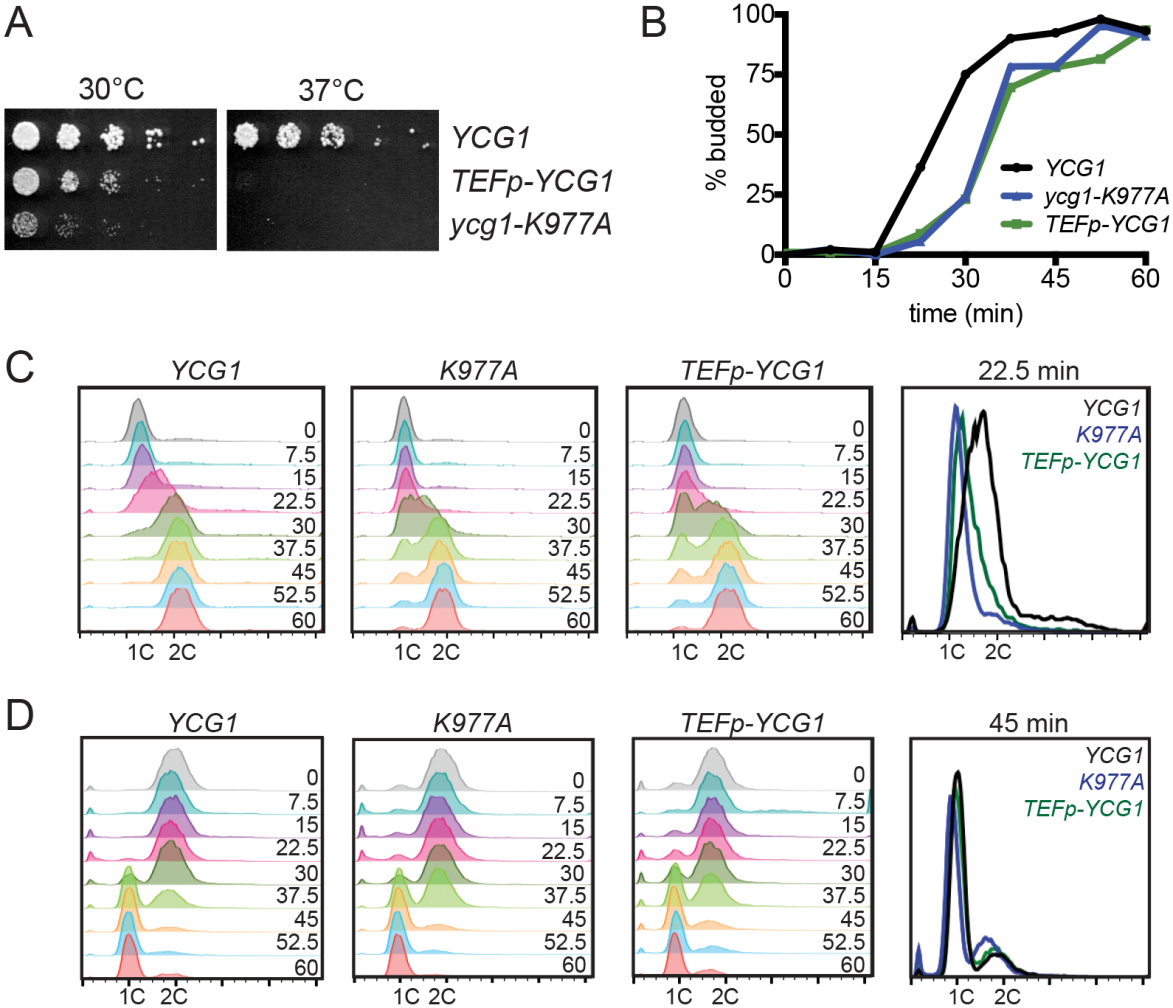
Increased expression of Ycg1 delays entry into S-phase. **(A)** 5-fold dilutions of strains with the indicated genotypes (YTD33, YTD148, YTD336) were plated on YPD and incubated at the indicated temperatures until the colonies in the wild-type strain were of similar size. **(B-C)** Wild-type (YTD276), *ycg1-K977A* (YTD290), and *TEFp-YCG1* (YTD361) strains were synchronized in G1 as in Fig 4D and samples were fixed at 7.5 minute intervals after release to measure the percentage of budded cells (B), and progression through S phase by flow cytometry (C). **(D)** Wild-type (YTD276), *ycg1-K977A* (YTD290), and *TEFp-YCG1* (YTD361) strains were synchronized in metaphase with a *MET3p-CDC20* shut-off allele then released into the cell cycle and samples fixed at 7.5 minute intervals after release. Progression into G1 phase was measured by flow cytometry.

To distinguish between these effects and determine whether the increased expression of Ycg1-K977A was the primary cause of the proliferation defects described above, we disrupted cell cycle-regulation of Ycg1 levels in an alternative way, using the constitutive *TEF1* promoter to express Ycg1 at elevated levels throughout the cell cycle (Fig 4C and 4D). *TEFp-YCG1* strains showed no alteration in rDNA stability or silencing, confirming that *YCG1* overexpression does not impair condensin function (S2B Fig). Importantly, *TEFp-YCG1* strains displayed an increase in doubling time, similar to *ycg1-K977A* strains (Fig 4C). Furthermore, both *ycg1-K977A* and *TEFp-YCG1* strains showed a delay in G1/S progression (Fig 4B and 4D), and exhibited sensitivity to temperature stress (Fig 5A). These data argue that increasing Ycg1 abundance is sufficient to delay the cell cycle and decrease proliferation rate. Notably, overexpression of Ycg1 did not result in heterogeneous colony size or sensitivity to HU or benomyl (S2 Fig), which suggests that these phenotypes of the *ycg1-K977A* strain may result from its reduced function, and not increased expression of the stable protein.

The delay in cell-cycle progression described above could be the result of a delay in the G1/S transition and/or an inhibition of DNA replication in mutant strains. To determine whether the transition from G1 into S phase was delayed, we monitored budding, since bud formation is triggered by the wave of transcription that occurs at the G1/S transition, but is independent of replication initiation [46]. Interestingly, the delay in DNA synthesis in *ycg1-K977A* and *TEFp-YCG1* strains correlated with a proportional delay in budding (Fig 5B and 5C), indicating that these strains exhibit a delay in entering S phase. The delay was most evident 22.5 minutes after release from G1, when wild-type cells were in S phase and *ycg1-K977A* and *TEFp-YCG1* strains were largely still in G1 (Fig 5C). Consistent with a previous report [47], this delay was not observed in the condensin temperature-sensitive mutants *ycg1-2* and *brn1-9* [48] when they were released from G1 arrest at the restrictive temperature (S3 Fig), confirming that the G1/S delay observed upon Ycg1 overexpression is distinct from condensin loss of function.

Chromosomes decondense in telophase, so condensin activity must decrease at the end of mitosis. One possibility is that the increased Ycg1 levels in *ycg1-K977A* and *TEFp-YCG1* strains might impair chromatin decondensation, which could induce an additional cell-cycle delay when cells exit from mitosis. We tested for this possibility by synchronizing cells in metaphase with a *CDC20* shut-off allele and monitoring progression of each strain into G1 phase by flow cytometry. Although it is possible that the strains may progress through the stages of mitosis with slightly different kinetics, both strains entered G1 phase with similar timing to a wild-type strain (Fig 5D), suggesting that neither strain has a delay in exiting from mitosis. We also assayed chromosome condensation directly, by examining the structure of the rDNA locus, which undergoes compaction during mitosis that can be visualized in chromosome spreads [7,22,49]. Cells were arrested in both metaphase and G1, the rDNA was visualized by DAPI and Net1 staining of chromosome spreads, and condensation scored as previously described [22,47]. Notably, there was no significant difference in rDNA conformation between wild-type and *TEFp-YCG1* strains, in either metaphase or G1-arrested cells (S4 Fig). Together, these results argue that increasing Ycg1 expression does not alter rDNA condensation, or delay exit from mitosis.

### Ycg1 levels are limiting for condensin recruitment to chromatin

Our comparison of the expression levels of condensin subunits indicates that Ycg1 is expressed at lower levels than the other subunits (Fig 2A, S5A Fig). In addition, Ycg1 is the only condensin subunit that cycles (Fig 1C). These findings raise the possibility that Ycg1 levels might be limiting for complex formation. If this is the case, then overexpression of other subunits of the complex should not impair cell-cycle progression in the way that overexpression of Ycg1 does. To test this hypothesis, we integrated the *TEF1* promoter upstream of the other four subunits of the condensin complex. Importantly, although each condensin subunit was overexpressed in these strains to similar levels (S5A Fig), increasing expression of no other condensin subunit led to an increase in doubling time (Fig 4C). Moreover, while asynchronous *TEFp-YCG1* cells displayed an increased fraction of cells in G1 phase, consistent with a G1/S delay, there was no change in the fraction of G1 cells upon overexpression of any other condensin subunit (S5B Fig). These data are in agreement with the model that Ycg1 is the limiting subunit for condensin function.

**Fig 6:**
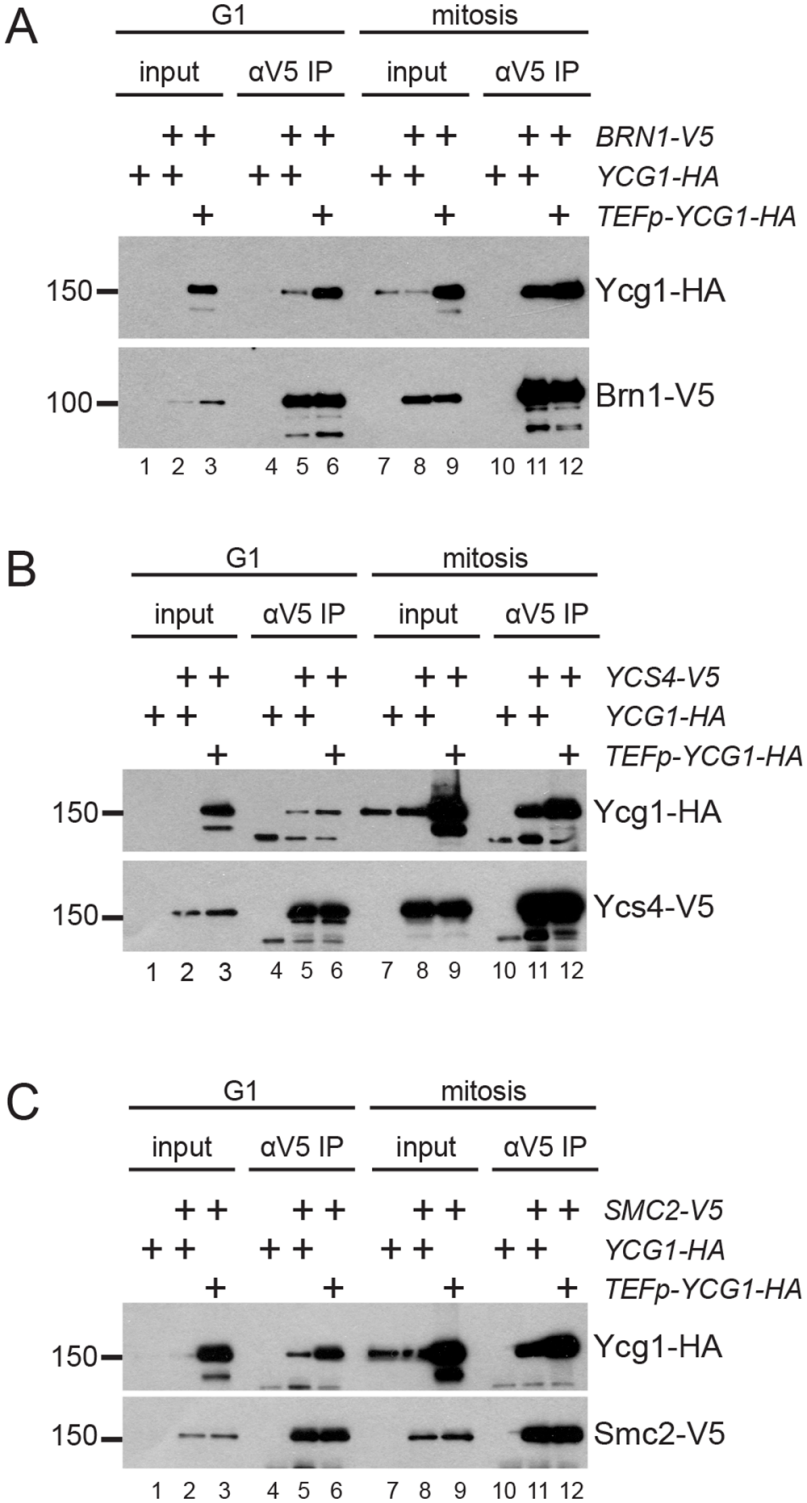
Cell cycle-regulation of Ycg1 limits condensin complex formation in G1 phase. *YCG1* (YTD302, YTD394, YTD396) and *TEFp-YCG1* (YTD355, YTD395, YTD397) strains were synchronized in mitosis with a *MET3p-CDC20* shut-off allele, or in G1 by releasing from the mitotic arrest into medium containing alpha-factor for 2 hours. Condensin complexes were then immunoprecipitated from arrested cells with antibodies against a 3V5 tag on Brn1 **(A)**, Ycs4 **(B)**, or Smc2 **(C)**, and Ycg1 association with each subunit was assayed by Western blot for the 3HA tag on Ycg1. In all experiments, a strain lacking a V5 tag (YTD276) was used as a negative control. Flow cytometry data verifying cell-cycle arrest is shown in S6 Fig.

Ycg1 has not been shown to function on its own, or as part of any protein complex other than condensin. Therefore, we hypothesized that increased Ycg1 expression slowed G1/S progression as a result of increased condensin complex during G1 phase. Notably, this hypothesis makes two predictions: first, that the amount of intact condensin complex varies based on cell-cycle position, and second, that modulation of Ycg1 levels is necessary to establish this variation. To test these possibilities, we assayed for changes in condensin subunit interactions in different cell-cycle phases. First, we arrested cells in G1 phase or mitosis, immunoprecipitated different subunits of the condensin complex, and determined whether more Ycg1 associated with each subunit in mitosis than in G1 phase. Importantly, more Ycg1 co-immunoprecipitated with other condensin subunits in mitosis than G1 (Fig 6, compare lanes 5 and 11 in each panel), confirming the level of intact condensin complex varies in different cell-cycle phases.

We simultaneously performed co-immunoprecipitation experiments in *TEFp-YCG1* strains to determine if preventing the downregulation of Ycg1 led to an increase in the amount of intact condensin complex. Notably, more Ycg1 was associated with other subunits of the complex in the *TEFp-YCG1* background compared to wild-type cells in G1 phase (Fig 6, compare lanes 5 and 6 in each panel). In mitotic cells we observed a small increase in Ycg1 interaction over the already high levels in wild-type cells when Ycg1 was overexpressed (Fig 6, compare lanes 11 and 12 in each panel). These data show that overexpression of Ycg1 increases condensin subunit interactions considerably in G1, when Ycg1 is limiting, and less so during mitosis, when Ycg1 levels peak.

**Fig 7:**
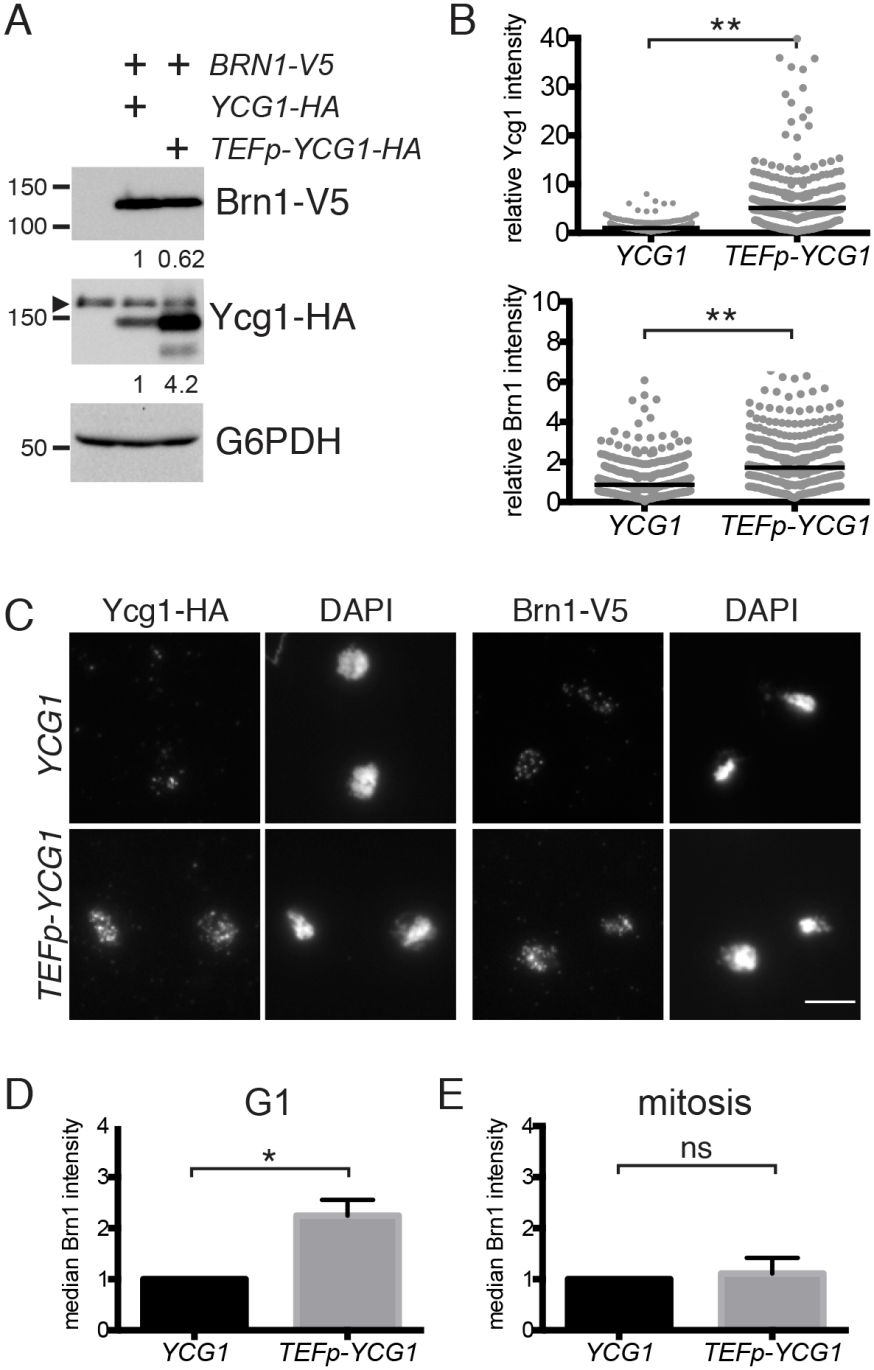
Ycg1 is limiting for condensin recruitment to chromatin. **(A)** Western blot showing total levels of Ycg1-3HA and Brn1-3V5 proteins in asynchronous wild-type (YTD297) and *TEFp-YCG1* (YTD342) cells. G6PDH is shown as a loading control. Relative expression of Ycg1 and Brn1, normalized to G6PDH, are indicated beneath each blot. **(B)** Representative experiment showing quantification of Ycg1-3HA and Brn1-3V5 staining of chromosome spreads from cells in (A). At least 190 cells in each sample were quantified, with the median intensity indicated by the black line. Values are shown relative to the median intensity in wild-type cells. Both proteins are significantly enriched on chromatin in *TEFp-YCG1* cells, as determined by an unpaired t-test (**p<0.0001). **(C)** Representative images of Ycg1-3HA and Brn1-3V5 staining on chromosome spreads from the experiment shown in (B). Scale bar represents 5 μm. **(D)** Wild-type (YTD297) and *TEFp-YCG1* (YTD342) cells were arrested in G1 with alpha-factor and chromosome spreads performed as in (B). Shown is the average of the median intensity values (normalized to the median in wild-type) from 3 experiments, +/- 1 standard deviation. Brn1 is significantly enriched on chromatin in *TEFp-YCG1* cells, as determined by an unpaired t-test (*p<0.05). **(E)** Brn1-3V5 staining on chromosome spreads as in (D) except strains were arrested in nocodazole. An unpaired t-test was used to confirm that there is no significant difference between strains (ns). Flow cytometry plots confirming cell-cycle positions for representative experiments are shown in S7 Fig.

A previous study demonstrated that Ycg1 is required to recruit other condensin subunits to chromatin [50]. Therefore, we investigated whether the chromatin association of the Brn1 subunit was increased in *TEFp-YCG1* cells by quantifying the amount of Brn1 that associated with chromosomes in a chromosome spread assay [7,17,51]. Notably, although overexpression of Ycg1 did not lead to increased levels of Brn1 (Fig 7A), the association of Brn1 with chromatin increased in *TEFp-YCG1* cells (Fig 7B and 7C). This increase in Brn1 association was observed in both asynchronous cells and cells arrested in G1 phase (Fig 7D). In contrast, there was no significant increase in bulk chromatin association of Brn1 in mitotic cells (Fig 7E). These results are consistent with the observation that increasing Ycg1 expression leads to a greater increase in the levels of intact complex in G1 than in mitosis (Fig 6), and support the possibility that an increase in the association of condensin with chromosomes in G1 phase delays cell-cycle entry.

**Fig 8:**
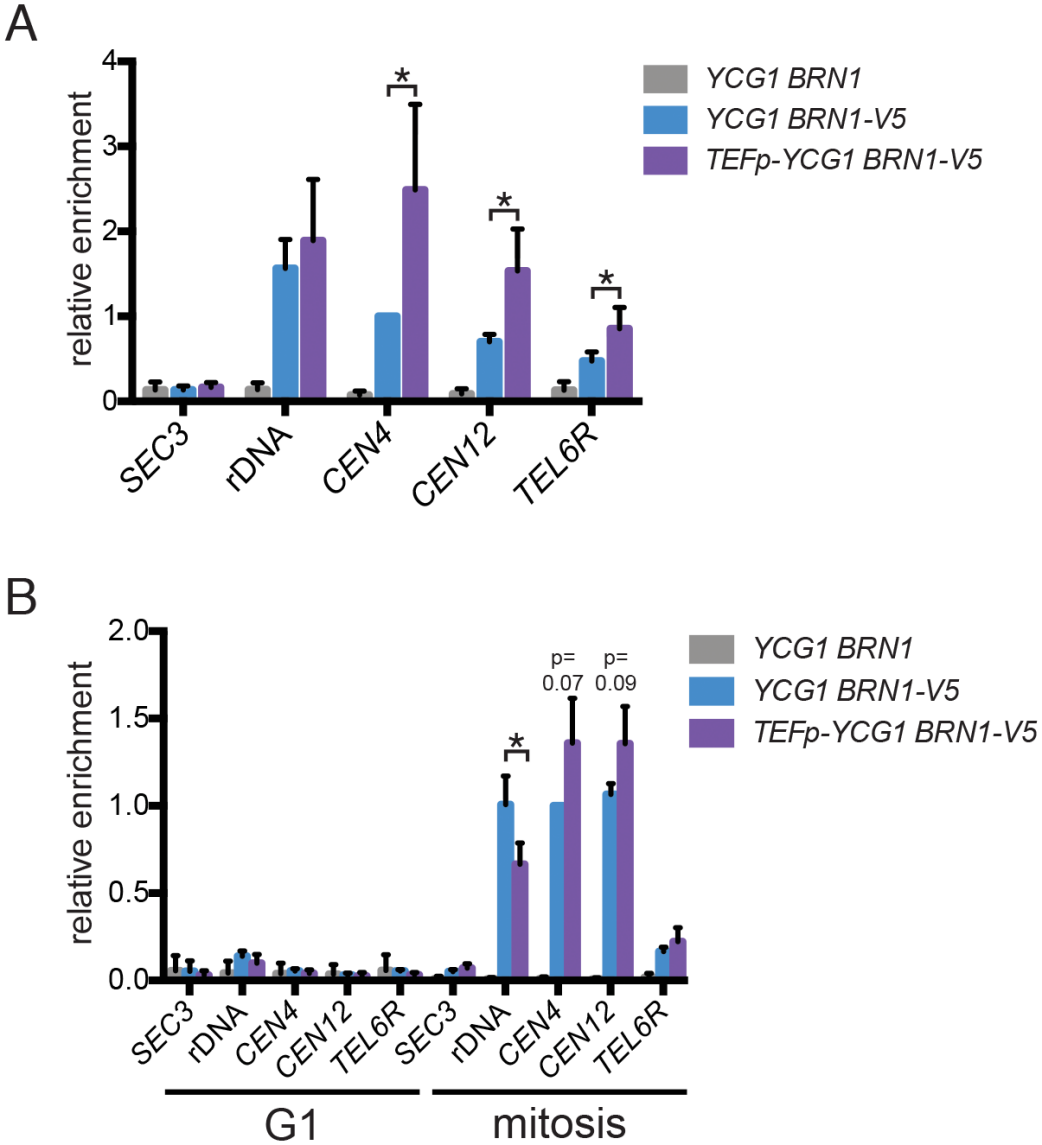
Increasing Ycg1 expression in G1 does not promote condensin recruitment to mitosis-enriched sites. **(A)** ChIP-qPCR of Brn1-3V5 from asynchronous *YCG1* (YTD297) and *TEFp-YCG1* (YTD342) strains, as well a wild-type strain lacking a 3V5 tag (YTD33) as a negative control. Brn1 enrichment at the rDNA (replication fork barrier), the centromeres of chromosome IV and XII (*CEN4* and *CEN12*), the subtelomeric region on the right arm of chromosome VI (*TEL6R*), and a condensin-depleted region in the *SEC3* gene on chromosome V (*SEC3*) was quantified by qPCR. Data was normalized to Brn1 enrichment at the *CEN4* locus in *YCG1 BRN1-3V5* cells in each experiment and represent mean values of 4 biological replicates +/- 1 standard deviation. Significance was determined by an unpaired t-test (*p<0.05). **(B)** ChIP-qPCR as in (A), except that strains were arrested in G1 with alpha-factor or in metaphase with nocodazole, prior to processing. Data was normalized to Brn1 enrichment at the *CEN4* locus in *YCG1 BRN1-3V5* cells arrested in mitosis in each experiment, and represent mean values of 3 biological replicates +/- 1 standard deviation. Significance was determined by an unpaired t-test (*p<0.05). For centromeric loci that did not show statistically significant increases in binding, the p-values are indicated. Flow cytometry plots confirming cell-cycle positions for all experiments are shown in S7 Fig.

Although condensin associates with chromosomes throughout the cell cycle, its enrichment at many of its best-characterized binding sites (including the rDNA, centromeres, and telomeres) is substantially higher in mitosis than in G1 [15,25,27,37,52]. Notably, each of these classes of binding sites requires mitosis-specific factors to stimulate this increase in condensin recruitment [24,27,53], which raises the question of whether or not Ycg1 overexpression leads to increased condensin binding to these specific loci during interphase. To address this question, we used chromatin immunoprecipitation and quantitative PCR (ChIP-qPCR) to quantify Brn1 recruitment to a representative set of these sites [16,17,27,53–55]. Interestingly, in asynchronous *TEFp-YCG1* cells, Brn1 binding increased at centromeric and telomeric loci, but not the rDNA (Fig 8A). The fact that condensin recruitment to the rDNA is largely unchanged in *TEFp-YCG1* strains is consistent with the fact that these cells do not show changes in rDNA condensation (S4 Fig), or in rDNA silencing or stability (S2B Fig).

We next used ChIP-qPCR to examine Brn1 recruitment to mitotic binding sites in cells that were arrested in G1 and metaphase, in order to directly compare binding at these sites to bulk chromatin binding that we had measured using chromosome spreads (Fig 7). These experiments led to two interesting observations. First, consistent with the results of the chromosome spread experiments, Brn1 binding to mitotic sites was not significantly elevated in metaphase cells upon overexpression of Ycg1 (Fig 8B). (Although binding at centromeres tended to be slightly elevated in *TEFp-YCG1* cells, the data did not reach statistical significance, and a modest reduction in binding to the rDNA was observed.) The second conclusion from these data is that although Brn1 bound to the rDNA, centromeres, and a telomere in metaphase, binding at each of these sites was reduced to background levels in both wild-type and *TEFp-YCG1* strains that were arrested in G1 (Fig 8B). This result indicates that although total Brn1 binding to chromosomes is elevated in *TEFp-YCG1* strains in G1 (Fig 7D), the complex is not enriched at mitosis-specific target sites. In addition, the increased binding of condensin to centromeres and telomeres that is seen in asynchronous *TEFp-YCG1* cells is likely to result from increased binding at a point in the cell cycle other than G1 or metaphase.

## Discussion

### Ycg1 limits condensin complex levels during interphase

Here, we show that cyclical transcription and proteasomal degradation regulate Ycg1 levels during the cell cycle, which in turn modulates condensin complex formation. Since Ycg1 is essential for condensin function [5–8], downregulation of its expression after mitosis (Fig 1) is predicted to reduce the amount of condensin complex and thereby decrease its association with chromosomes and activity. Indeed, we demonstrate that the amount of intact condensin complex is reduced in G1, concurrent with low Ycg1 expression, and increases during mitosis, when Ycg1 expression peaks (Figs 1 and 6). Our results also argue that Ycg1 levels are limiting, since overexpression of Ycg1 was sufficient to both increase complex formation (Fig 6) and recruitment to chromatin (Fig 7), as well as slow proliferation (Fig 5). In contrast, individual overexpression of the other four condensin subunits had no effect on proliferation rate (Fig 5A). Intriguingly, we found that the reduction in proliferation rate in *YCG1*-overexpressing cells was caused by a delay in progression through the G1/S transition (Fig 5D and 5E). These findings suggest that downregulation of Ycg1 is important to decrease condensin activity after mitosis, thereby allowing cells to proceed through interphase.

Although several studies have reported that *YCG1* mRNA is cell cycle-regulated [38–40], the question of whether or not Ycg1 protein cycles has not been addressed. Indeed, a prior study found that Ycg1 protein is expressed at lower levels in G1 than in S phase and mitosis [22], whereas others show a more constitutive expression pattern across the cell cycle [14,24]. For this reason, we analyzed the expression of Ycg1 in different strain backgrounds with different epitope tags (Fig 1B–1D). Importantly, in each case we found that Ycg1 cycled and mirrored the mRNA expression pattern (Fig 1). Furthermore, since disrupting cyclical expression of Ycg1 increased condensin complex formation and slowed proliferation (Figs 4A, 5A and 6), we conclude that cyclical expression of Ycg1 is functionally important for cell-cycle progression.

### An updated model for cell cycle-regulation of condensin

Previous studies have shown that phosphorylation of condensin subunits by mitotic kinases stimulates the supercoiling activity of the complex [13,14,22–24,37], suggesting that phosphorylation is one mechanism that helps restrict chromosome condensation to mitosis. In addition, recruitment of the complex to specific sites is known to be dependent upon mitosis-specific factors, such as Sgo1, which recruits condensin to centromeres in S phase through mitosis [26,27]. Our results reveal an additional regulatory mechanism that contributes to the reduction in condensin activity after mitosis is complete. Ycg1 levels limit the amount of condensin complex early in the cell cycle, and by extension reduce the amount of condensin that is available to act on chromatin. These findings suggest a revised model in which condensin complex formation, recruitment to a subset of binding sites, and phosphorylation are regulated to ensure that condensin activity is at its lowest level during G1 phase [13,14]. As cells progress through S phase into mitosis, Ycg1 levels rise, condensin complex formation increases, and more complex is loaded onto chromatin. Finally, during mitosis condensin is recruited to several mitosis-specific sites [25–27,37], and the complex is activated by mitotic kinases to increase its supercoiling activity [13,14]. Thus, complex formation, the availability of recruitment factors, and phosphorylation act together to establish different states of condensin activity in different cell-cycle stages.

One interesting possibility raised by our results is that constitutive expression of Ycg1 could disrupt the timing of chromosome condensation, or lead to precocious condensation of chromosomes early in the cell cycle. We tested this possibility by examining condensation of the rDNA, which undergoes the most dramatic condensin-dependent structural change during mitosis in yeast, but did not observe any change in condensation in *TEFp-YCG1* cells arrested in metaphase, or any increased condensation in cells arrested in G1 (S4 Fig). Consistent with this result, condensin binding to the rDNA did not increase upon Ycg1 overexpression (Fig 8); therefore, factors that promote mitotic enrichment of condensin at the rDNA are likely necessary to drive rDNA compaction. It remains possible that the timing of condensation is altered as cells enter or exit mitosis. Alternatively, precocious condensation could occur elsewhere in the genome. However, since the activating phosphorylations on the complex are absent G1 phase [14,24], a likely possibility is that excess condensin in G1 does not drive precocious condensation but instead binds to chromatin and increases interactions between distant sites in the genome, or physically blocks the chromatin association of transcription or replication factors.

Although cells that express stable Ycg1 (*ycg1-k977A*) and those that overexpress wild-type Ycg1 show similar delays in cell-cycle entry, we find that they respond differently to some cell-cycle perturbations. Notably, *ycg1-K977A* cells are sensitive to the replication inhibitor hydroxyurea (HU), whereas *TEFp-YCG1* cells are not (S2D Fig). HU sensitivity has been previously reported in fission yeast expressing a temperature-sensitive allele of the kleisin subunit of condensin, Cnd2 [45]. Thus, HU sensitivity is consistent with the possibility that the K977A mutation in Ycg1 partially impairs some aspect of condensin function, while still promoting an increase in complex levels in G1 phase that delays cell-cycle entry.

Stabilization and overexpression of Ycg1 also result in different responses to the microtubule poison benomyl, which activates the spindle assembly checkpoint (S2E Fig). This finding is intriguing because condensin has an established function at centromeres, where it promotes chromosome biorientation by biasing kinetochores for capture by microtubules from opposite poles [26,56]. Interestingly, although *ycg1-K977A* strains exhibit increased sensitivity to benomyl (consistent with a partial loss of function), cells overexpressing Ycg1 are more resistant to spindle disruption than wild-type cells. This raises the possibility that when Ycg1 is expressed at high levels early in the cell cycle, more condensin may be loaded at centromeres, which could enable cells to respond better to spindle disruption. Our data is consistent with this hypothesis. Although we did not observe a significant increase in condensin recruitment to centromeres in G1 or metaphase-arrested cells (Fig 8B), recruitment was increased in asynchronous *TEFp-YCG1* cells compared to wild-type (Fig 8A). In the future it will be interesting to examine the dynamics of condensin recruitment to centromeres during the cell cycle, in order to determine if condensin is recruited to centromeres earlier in S-phase when Ycg1 is overexpressed, or if it persists at centromeres longer as cells progress through mitosis.

### Limiting condensin subunits in other eukaryotes

Although our data shows that condensin is regulated by limiting expression of the Cap-G subunit in budding yeast, evidence suggests that similar mechanisms control the activity of condensin in other systems. Indeed, proteolytic regulation of condensin also occurs in *Drosophila melanogaster*, via targeting of the kleisin subunit of condensin II, Cap-H2 [32]. In that system, blocking Cap-H2 degradation results in increased chromosome condensation in interphase cells [32,33]. However, it remains to be determined if stable Cap-H2 can delay the G1/S transition, as Ycg1 stabilization does in yeast.

Notably, although Ycg1 and Cap-H2 are similarly regulated, they are not orthologs [11]. Indeed, we posit that the existence of a rate-limiting subunit of the condensin complex may have evolved independently in fungi and animals, with different subunits being targeted for degradation. Importantly, the presence of proteolytic regulation in two evolutionarily distant eukaryotes, and the interphase phenotypes observed when proteolysis is disrupted, suggests that this regulation may be an important mechanism to limit condensin function in all eukaryotes. Human condensin II-specific subunits are also reported to undergo proteolytic regulation [35,36], and in the future it will be of interest to determine whether any of these subunits are rate limiting in mammalian cells. Limiting the levels of a condensin subunit is a mechanism that is likely to coordinate changes in chromosome structure with cell-cycle stage in all eukaryotes, and may also have broader roles in modulating condensin activity in response to specific environmental signals.

## Materials and Methods

### Yeast strains

A complete list of strains used in this study can be found in S1 Table. All experiments were performed at 30°C, unless otherwise indicated. Strains were grown in rich medium with 2% dextrose, except for strains harboring *MET3p-CDC20*, which were grown in synthetic complete medium lacking methionine with 2% dextrose.

Epitope-tagging of genes was achieved by integrating 3HA-His3MX6, 3V5-kanMX6, or 13Myc-His3MX6 in place of the stop codon at the genomic locus of each gene, as indicated in S1 Table. To generate strains that could be synchronized in metaphase, the methionine-regulatable *MET3* promoter was integrated upstream of *CDC20* using plasmid pBO1105. pBO1105 is a modification of YIp22(*TRP1*) *MET3p-CDC20* [57] in which the YIp22 vector has been replaced with pAG25 (J.J. Li, personal communication). Where indicated, the *TEF1* promoter was integrated upstream of the start codons of condensin subunits, as previously described [58]. Mutations in *YCG1* were introduced into the genome by deleting the non-essential 3’ end of the gene, followed by integration of PCR products that replace the 3’ sequence and include the indicated mutations. All mutations were confirmed by sequencing. For proteasome inhibition experiments, Ycg1 was tagged in strain YUS5, which carries mutations that increase its sensitivity to proteasome inhibitors [59,60]. To assay silencing and recombination at the rDNA locus, *ycg1-K977A* and *TEFp-YCG1* were integrated into strain JS306 and strains were assayed as previously described [44]. To integrate an extra copy of *YCG1* at the *URA3* locus, *YCG1* (with 362 base pairs of its upstream sequence) was cloned into pRS306 and the resulting vector was digested with NcoI for integration at *URA3*. Single copy integration was confirmed by PCR. Strains expressing temperature-sensitive condensin alleles were previously described in [48].

### Cycloheximide-chase assays

To assay protein degradation, cycloheximide (50 μg/mL) was added to cells and samples taken after the indicated number of minutes. At each time point equivalent optical densities of cells were collected. To assay stability upon proteasome inhibition, cells were grown in synthetic complete medium lacking proline with 0.003% SDS and 2% dextrose, then treated with DMSO or 5 μg/ml MG132 for 2 hours prior to the addition of cycloheximide. Where indicated, cells were arrested with 10 μg/ml alpha-factor for 2.5 hours, or 10 μg/ml nocodazole for 2 hours, before the addition of cycloheximide. In all experiments cell-cycle arrest was verified by flow cytometry.

### Western blotting

Samples were prepared for Western Blotting by resuspending equivalent optical densities of cells in preheated SDS sample buffer (50 mM Tris pH 7.5, 5 mM EDTA, 5% SDS, 10% glycerol, 0.5% β-mercaptoethanol, bromophenol blue, 1 μg/ml leupeptin, 1 μg/ml bestatin, 1 mM benzamidine, 1 μg/ml pepstatin A, 17 μg/ml PMSF, 5 mM sodium fluoride, 80 mM β-glycerophosphate and 1 mM sodium orthovanadate), followed by incubation at 95°C for 5 minutes. Glass beads were then added and samples were bead beat using a Biospec Mini-Beadbeater for 3 minutes. Samples were clarified by centrifugation and analyzed by SDS-PAGE followed by Western blotting. Western blots were carried out with antibodies against GFP (clone JL-8, Clontech), Clb2 (y-180, Santa Cruz Biotechnology), Cdc28/Cdk1 (yC-20, Santa Cruz Biotechnology), HA (clone 12CA5), V5 (ThermoFisher), Myc (clone 9E10, Covance), and G6PDH (Sigma). Where indicated, quantitation was performed using a BioRad ChemiDoc Touch imaging system and the accompanying ImageLab software.

### Cell cycle arrest

G1 cell-cycle arrest was achieved by incubating logarithmic-phase cells with 10 μg/ml alpha-factor for 2–3 hours, as indicated. Mitotic arrest was achieved by treating cells with 10 or 20 μg/ml nocodazole for 2–3 hours, or by adding 5X L-methionine (0.1 mg/L final concentration) to *MET3p-CDC20* strains (growing in medium without methionine) for 3.5 hours. Where indicated *MET3p-CDC20* strains were arrested in mitosis as above, then released into medium without methionine containing alpha-factor for 2.5 hours to synchronize cells in G1, followed by release into medium without methionine or alpha-factor. Details of specific arrest-release experiments are indicated in the figure legends.

### Cell cycle analysis

Cell-cycle positions were confirmed by flow cytometry. Cells were fixed and labeled with Sytox Green (Invitrogen) as previously described [61]. Samples were analyzed using a FACScan (Becton Dickinson) and data analyzed with FlowJo (Tree Star, Inc.) software. Where indicated, fixed cells were sonicated and percentage of budded cells determined by counting at least 100 cells/sample.

### rDNA silencing and stability assays

rDNA silencing and stability were assayed in strains derived from JS306, as previously described [44]. In these strains, two PolII-regulated marker cassettes are integrated into different rDNA repeats: a single *MET15* reporter gene (embedded in a Ty1 element) is integrated within NTS2 of one rDNA repeat, and a *mURA3/HIS3* expression cassette is integrated within the 18S rRNA-coding region of a second repeat.

In this assay, the *MET15* reporter is used to score an increase in recombination between rDNA repeats. The expression of *MET15* results in white colonies on MLA plates (Pb+ plates), loss of the *MET15* gene results in dark brown colonies or sectors (as seen in the *sir2Δ* strain), and if the *MET15* gene is present, but is silenced, the colonies are a tan color. Strains are scored as having increased recombination between rDNA repeats if dark brown and sectored colonies are observed on MLA plates, which indicates loss of the *MET15* gene. Although a tan color indicates *MET15* gene is present, but silenced, the shade of tan is variable between experiments and therefore not used to infer the degree of silencing.

In the same strains the *mURA3/HIS3* reporter is used to assay silencing. Strains that are capable of silencing do not express *mURA3* and thus can’t grow on—Ura plates, however *HIS3* is incompletely silenced so strains can grow on—His plates. For this reason, growth on—His is used as a confirmation that the strains retain the *mURA3/HIS3* cassette. Strains that grow similarly on—His and—Ura plates are scored as having a loss of silencing of the rDNA locus. *sir2Δ* mutants were previously shown to have both decreased silencing and increased recombination [44], and serve as a positive control for both readouts.

### Co-immunoprecipitation assays

Cell pellets from 30 optical densities of arrested cells were lysed by resuspension in HEPES lysis buffer (25mM HEPES-OH pH 7.5, 250mM NaCl, 0.2% Triton, 1mM EDTA, 10% glycerol, 1 μg/ml leupeptin, 1 μg/ml bestatin, 1 mM benzamidine, 1 μg/ml pepstatin A, 17 μg/ml PMSF, 5 mM sodium fluoride, 80 mM β-glycerophosphate and 1 mM sodium orthovanadate), followed by 3 cycles of bead-beating for one minute each (with 5 minute incubations on ice between cycles). Protein concentrations were measured by Bradford assay and equal amounts of total protein were incubated with 2μL mouse anti-V5 antibody (ThermoFisher) for 3 hours, followed by addition of 25μL protein G magnetic beads (NEB) for 1 hour. Beads were washed 3X with HEPES lysis buffer and proteins were eluted by boiling in 2X sample buffer.

### Doubling time analysis

Cultures were grown to logarithmic phase, then diluted to 0.1 optical densities and 100μL of each was added in triplicate to a round bottom 96-well plate. Cell proliferation was monitored by growing cultures at 30°C with shaking in a Tecan Infinite M200 Pro plate reader and measuring optical density at 600nM every 20 minutes until cultures reached approximately 0.8 OD. Doubling times were calculated by fitting data points between 0.15 OD and 0.6 OD to an exponential growth equation using GraphPad Prism software.

### Chromosome spreads

Chromosome spreads to analyze condensin association with chromatin and rDNA morphology were performed as previously described [7,17,51]. 3HA-tagged Ycg1 was detected with mouse anti-HA antibody (clone 12CA5), 3V5-tagged Brn1 and 3V5-tagged Net1 were detected with mouse anti-V5 antibody (ThermoFisher), all in combination with Alexa Fluor 488-conjugated goat anti-mouse IgG (ThermoFisher) and DAPI. A wild-type strain lacking both epitope tags was used as a negative control in all experiments. To quantify Ycg1 and Brn1 chromatin binding, Alexa Fluor 488 fluorescence intensities within an area encompassing the merged Alexa Fluor and DAPI images were measured after background subtraction in ImageJ software. At least 190 cells were quantified for each sample, in each experiment. To score condensation of the rDNA, the rDNA structure (evident both by Net1 staining and the conformation of the DAPI-stained nucleolar DNA) in at least 200 cells were classified as either puffs (decondensed) or loop/lines (condensed), as previously described [7,22,49]. For all chromosome spreads performed on synchronized cultures, cells were first arrested with 10μg/ml alpha-factor or 20μg/ml nocodazole for 3 hours.

### Chromosome immunoprecipitation

Chromosome immunoprecipitation (ChIP) was performed as previously described [37] with the following modifications. For asynchronous and nocodazole-arrested cultures, 40 optical densities (ODs) of each culture were lysed in a Mini-Beadbeater (Biospec) and lysates were sonicated using a Diagenode Biorupter. For alpha-factor arrested cultures, 70 OD were used. Brn1-3V5 was immunoprecipitated with mouse anti-V5 (ThermoFisher) coupled to Protein G magnetic beads (New England Biolabs). Eluted DNA was quantified by qPCR on an Eppendorf Realplex system. Primers used for qPCR are listed in S2 Table.

## Supporting Information

S1 Fig Summary of Ycg1 C-terminal mutations and their effects on Ycg1 stability. **(A)** Diagram of all Ycg1 mutations tested. Stability of each mutant was assayed by cycloheximide-chase assay: (+) protein is degraded similar to wild-type, (+/-) modest stabilization compared to wild-type Ycg1, and (-) protein does not get degraded. **(B)** Cycloheximide-chase assay (left) and doubling time analysis (right) of strains expressing wild-type Ycg1 (YTD33), or proteins that harbor mutations in putative phosphorylation sites or acidic amino acids (YTD128, YTD199, YTD176). Mutation of threonine and serine residues results in a modest increase in stability, whereas mutation of acidic residues has no effect on protein turnover. Neither mutant increases the doubling time of cells.
(TIF)

S2 Fig Analysis of *ycg1-K977A* and *TEFp-YCG1* strains. **(A)** Heterozygous diploid cells expressing one allele of 3V5-tagged Ycg1 or Ycg1-K977A, as well as one allele of 13Myc-tagged Ycs4 (YTD284, YTD268, YTD269), Smc2 (YTD274, YTD285, YTD267), or Smc4 (YTD275, YTD286, YTD255) were used to assay condensin complex formation. Ycg1 was immunoprecipitated via its 3V5 tag in each strain, and the association of each other tagged subunit assayed by Western blot against the Myc tag. Ycg1-K977A associates with each other subunit as well as wild-type Ycg1. **(B)** rDNA silencing and stability were assayed using previously described strains that harbor multiple markers integrated into the rDNA locus [44]. Wild-type (YHA212), *ycg1-K977A* (YHA214), and *TEFp-YCG1* (YHA215) strains were compared to a *sir2Δ* strain (JS576) previously shown to have a silencing defect and to exhibit increased recombination at the rDNA locus [44]. In this assay, a silencing defect is detected by growth on—Ura plates and increased rDNA recombination is evident by dark brown and/or sectored colonies on MLA plates. Growth on—His plates confirms the presence of the *mURA3*/*HIS3* cassette. Stabilization or overexpression of *YCG1* does not result in either phenotype, confirming there is no defect in rDNA regulation in these strains. **(C)** Wild type (YTD33), *ycg1-K977A* (YTD148) and *TEFp-YCG1* (YTD336) strains were grown on YPD plates. Images show representative colony sizes. **(D)** Strains from (C) were diluted five-fold and spotted onto YPD plates, or YPD plates containing 100mM hydroxyurea (HU), and incubated at 30°C for the indicated number of days. **(E)** Strains from (C) were diluted 5-fold and spotted onto YPD plates, or YPD plates containing the indicated concentrations of benomyl. Notably, *TEFp-YCG1* cells exhibit resistance to high concentrations of benomyl, which could result from an increase in condensin association with centromeres in this strain (Fig 8A). **(F)** An extra copy of *YCG1* expressed from its own promoter was integrated into the *URA3* locus in the *ycg1-K977A* strain. The doubling time of the resulting strain (YTD430) was compared to the parental strains (YTD148) and a wild-type strain (YTD33). Shown is the average doubling time from three independent experiments +/- 1 standard deviation.
(TIF)

S3 Fig Inactivation of condensin does not delay the G1/S transition. **(A-B)** Wild type (MW836a), *ycg1-2* (Y10100), and *brn1-9* (Y9804) strains were arrested in G1 with alpha-factor for 4 hours at 23°C (with additional alpha-factor added after 2 hours) and released into fresh medium without alpha-factor at 34°C. Samples were fixed every 10 minutes for 60 minutes following release. DNA replication was monitored by flow cytometry (A), and number of budded cells counted (B), at each time point. **(C)** 5-fold dilutions of the strains from (A) were plated on YPD plates and incubated at the indicated temperatures. Both *ycg1-2* and *brn1-9* strains arrest at 34°C.
(TIF)

S4 Fig Analysis of rDNA condensation upon *YCG1* overexpression. **(A)** Representative images of rDNA morphology as visualized by chromosome spreads. Cells were arrested in G1 by the addition of alpha-factor, or in metaphase by the addition of 20μg/ml nocodazole, for 3 hours. Spheroplasts were prepared and chromosomes spread on glass slides. Chromosomes were stained with DAPI and the rDNA was visualized by immunofluorescence to detect 3V5-tagged Net1, which is enriched on nucleolar DNA [22,47]. A puff represents decondensed DNA, whereas a loop represents condensed rDNA. **(B)** Percentages of rDNA puffs and loops/lines in wild-type (YJB653) and *TEFp-YCG1* (YJB651) cells arrested in metaphase. In each experiment at least 130 cells were scored. Shown are the mean percentages +/- 1 standard deviation from n = 4 (YJB653) and n = 3 (YJB651) experiments. An unpaired t-test was used to confirm that there is no statistically significant difference between strains. **(C)** Percentages of rDNA puffs and loops/lines in wild-type (YJB653) and *TEFp-YCG1* (YJB651) cells arrested in G1. In each experiment at least 100 cells were scored. Shown are the mean percentages +/- 1 standard deviation from n = 4 (YJB653) and n = 3 (YJB651) experiments. An unpaired t-test was used to confirm that there is no statistically significant difference between strains.
(TIF)

S5 Fig Expression of condensin subunits in *TEF1* promoter knock-in strains. **(A)** Western blot showing relative expression of each 3HA-tagged condensin subunit in asynchronous wild-type (YTD33, YTD82, YTD83, YTD84, YTD80) and *TEF1p* knock-in (YTD336, YTD337, YTD353, YTD349, YTD362) strains. Cdk1 is shown as a loading control. **(B)** DNA content of asynchronous cultures as measured by flow cytometry showing the cell-cycle distributions of asynchronous cultures of the strains from (A). Note that overexpression of Ycg1, but not any other condensin subunit, results in a larger fraction of cells in G1 phase, consistent with a G1/S delay.
(TIF)

S6 Fig Flow cytometry data to support Fig 6. **(A-C)** G1 and mitotic arrests were confirmed by flow cytometry for the experiments shown in Fig 6A–6C, respectively.
(TIF)

S7 Fig Flow cytometry data to support Figs 7 and 8. **(A)** DNA content analysis of asynchronous cells (YTD297, YTD342) from the chromosome spread assay shown in Fig 7. Note that there is a smaller fraction of cells in mitosis in the *TEFp-YCG1* strain, so the increase in Ycg1 and Brn1 association with chromatin in this strain (Fig 7B and 7C) is not due to an increase in the number of mitotic cells. **(B)** DNA content analysis to confirm G1 arrest from a representative experiment included in Fig 7D. **(C)** DNA content analysis to confirm mitotic arrest from a representative experiment included in Fig 7E. **(D)** DNA content analysis to show cell cycle distributions from a representative experiment included in Fig 8A. **(E)** DNA content analysis to confirm G1 and mitotic arrest from a representative experiment included in Fig 8B.
(TIF)

S1 Table Strains list. (PDF)

S2 Table qPCR primer list. (PDF)
