# Supplementary figures and images for: Levels of Ycg1 Limit Condensin Function during the Cell Cycle
Source: PLoS Genet. 2016 Jul 27;12(7):e1006216. doi: 10.1371/journal.pgen.1006216 (PMC4963108; doi:10.1371/journal.pgen.1006216)

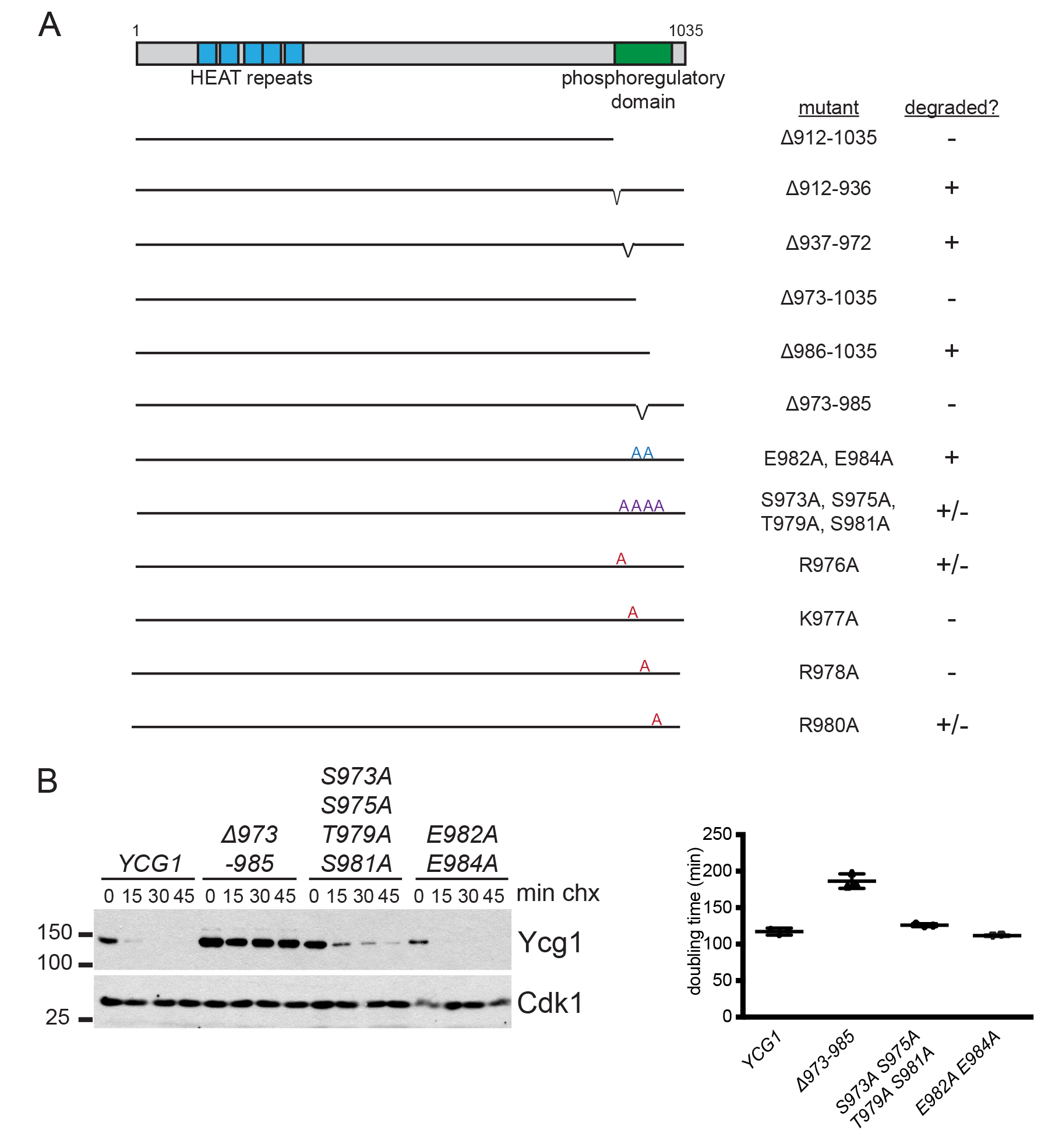

Supplement: S1 Fig — (A) Diagram of all Ycg1 mutations tested. Stability of each mutant was assayed by cycloheximide-chase assay: (+) protein is degraded similar to wild-type, (+/-) modest stabilization compared to wild-type Ycg1, and (-) protein does not get degraded. (B) Cycloheximide-chase assay (left) and doubling time analysis (right) of strains expressing wild-type Ycg1 (YTD33), or proteins that harbor mutations in putative phosphorylation sites or acidic amino acids (YTD128, YTD199, YTD176). Mutation of threonine and serine residues results in a modest increase in stability, whereas mutation of acidic residues has no effect on protein turnover. Neither mutant increases the doubling time of cells. (TIF) [file pgen.1006216.s001.tif]

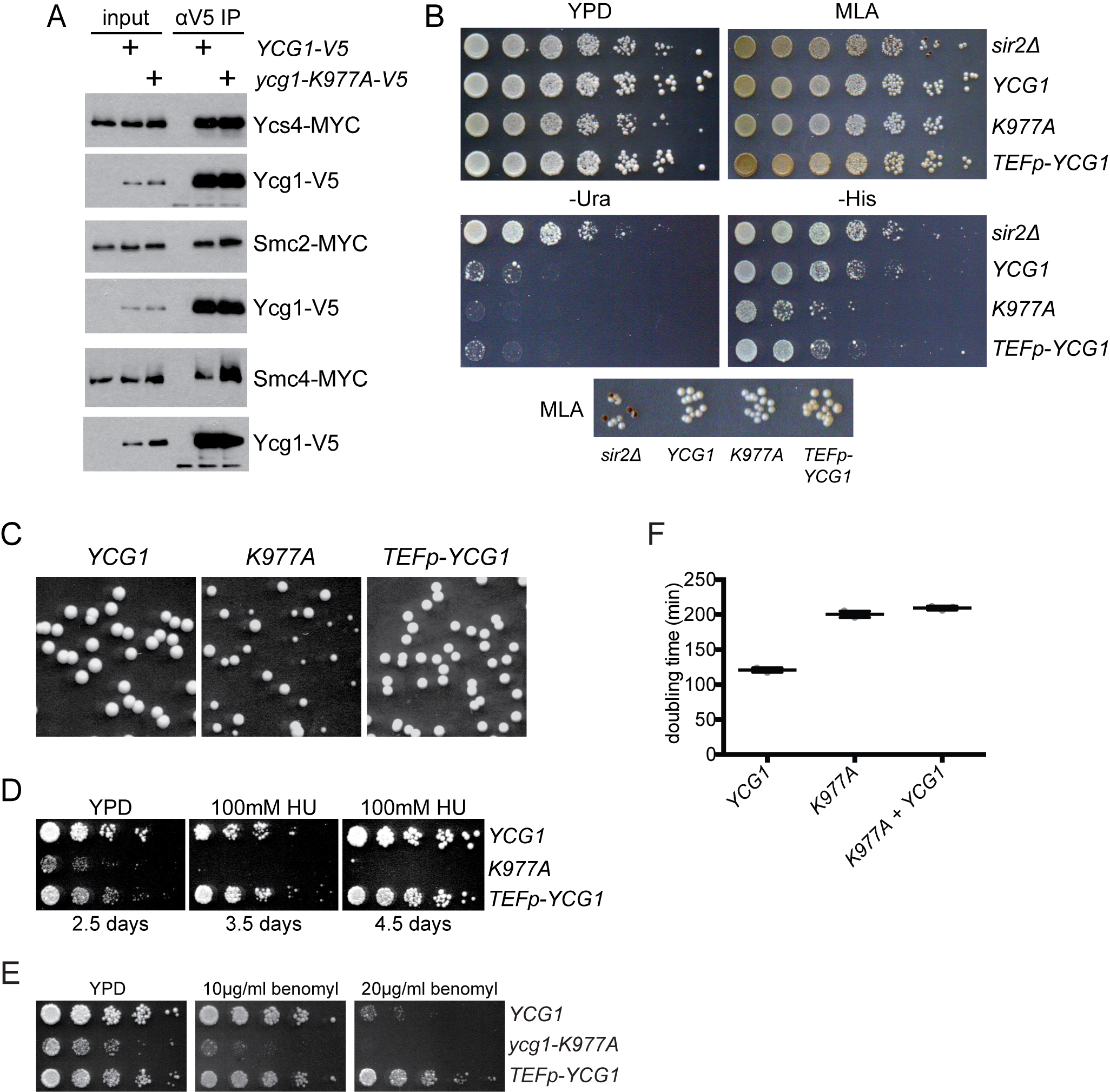

Supplement: S2 Fig — (A) Heterozygous diploid cells expressing one allele of 3V5-tagged Ycg1 or Ycg1-K977A, as well as one allele of 13Myc-tagged Ycs4 (YTD284, YTD268, YTD269), Smc2 (YTD274, YTD285, YTD267), or Smc4 (YTD275, YTD286, YTD255) were used to assay condensin complex formation. Ycg1 was immunoprecipitated via its 3V5 tag in each strain, and the association of each other tagged subunit assayed by Western blot against the Myc tag. Ycg1-K977A associates with each other subunit as well as wild-type Ycg1. (B) rDNA silencing and stability were assayed using previously described strains that harbor multiple markers integrated into the rDNA locus [44]. Wild-type (YHA212), ycg1-K977A (YHA214), and TEFp-YCG1 (YHA215) strains were compared to a sir2Δ strain (JS576) previously shown to have a silencing defect and to exhibit increased recombination at the rDNA locus [44]. In this assay, a silencing defect is detected by growth on—Ura plates and increased rDNA recombination is evident by dark brown and/or sectored colonies on MLA plates. Growth on—His plates confirms the presence of the mURA3/HIS3 cassette. Stabilization or overexpression of YCG1 does not result in either phenotype, confirming there is no defect in rDNA regulation in these strains. (C) Wild type (YTD33), ycg1-K977A (YTD148) and TEFp-YCG1 (YTD336) strains were grown on YPD plates. Images show representative colony sizes. (D) Strains from (C) were diluted five-fold and spotted onto YPD plates, or YPD plates containing 100mM hydroxyurea (HU), and incubated at 30°C for the indicated number of days. (E) Strains from (C) were diluted 5-fold and spotted onto YPD plates, or YPD plates containing the indicated concentrations of benomyl. Notably, TEFp-YCG1 cells exhibit resistance to high concentrations of benomyl, which could result from an increase in condensin association with centromeres in this strain (Fig 8A). (F) An extra copy of YCG1 expressed from its own promoter was integrated into the URA3 locus in the ycg1-K977A strain. [file pgen.1006216.s002.tif]

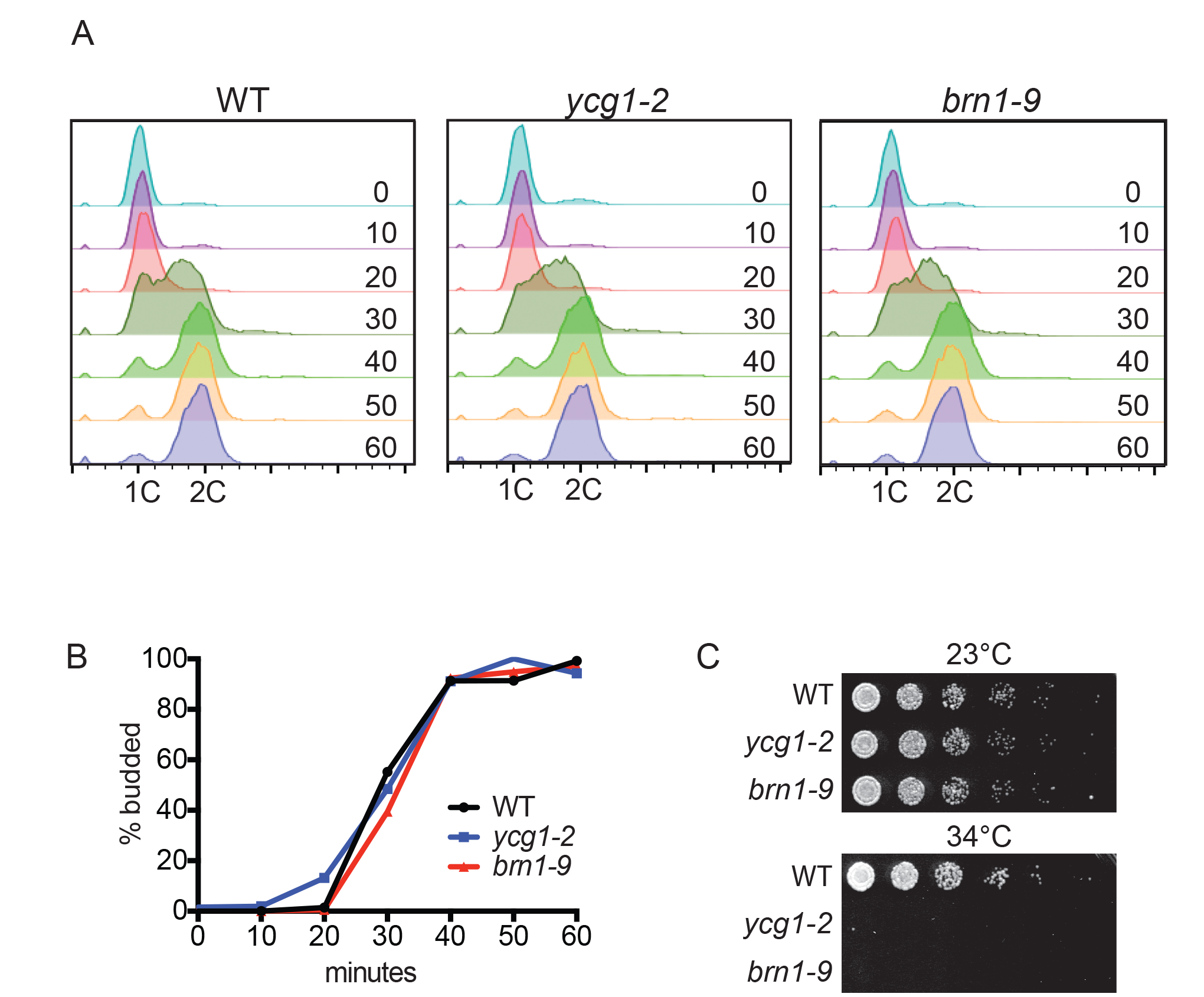

Supplement: S3 Fig — (A-B) Wild type (MW836a), ycg1-2 (Y10100), and brn1-9 (Y9804) strains were arrested in G1 with alpha-factor for 4 hours at 23°C (with additional alpha-factor added after 2 hours) and released into fresh medium without alpha-factor at 34°C. Samples were fixed every 10 minutes for 60 minutes following release. DNA replication was monitored by flow cytometry (A), and number of budded cells counted (B), at each time point. (C) 5-fold dilutions of the strains from (A) were plated on YPD plates and incubated at the indicated temperatures. Both ycg1-2 and brn1-9 strains arrest at 34°C. (TIF) [file pgen.1006216.s003.tif]

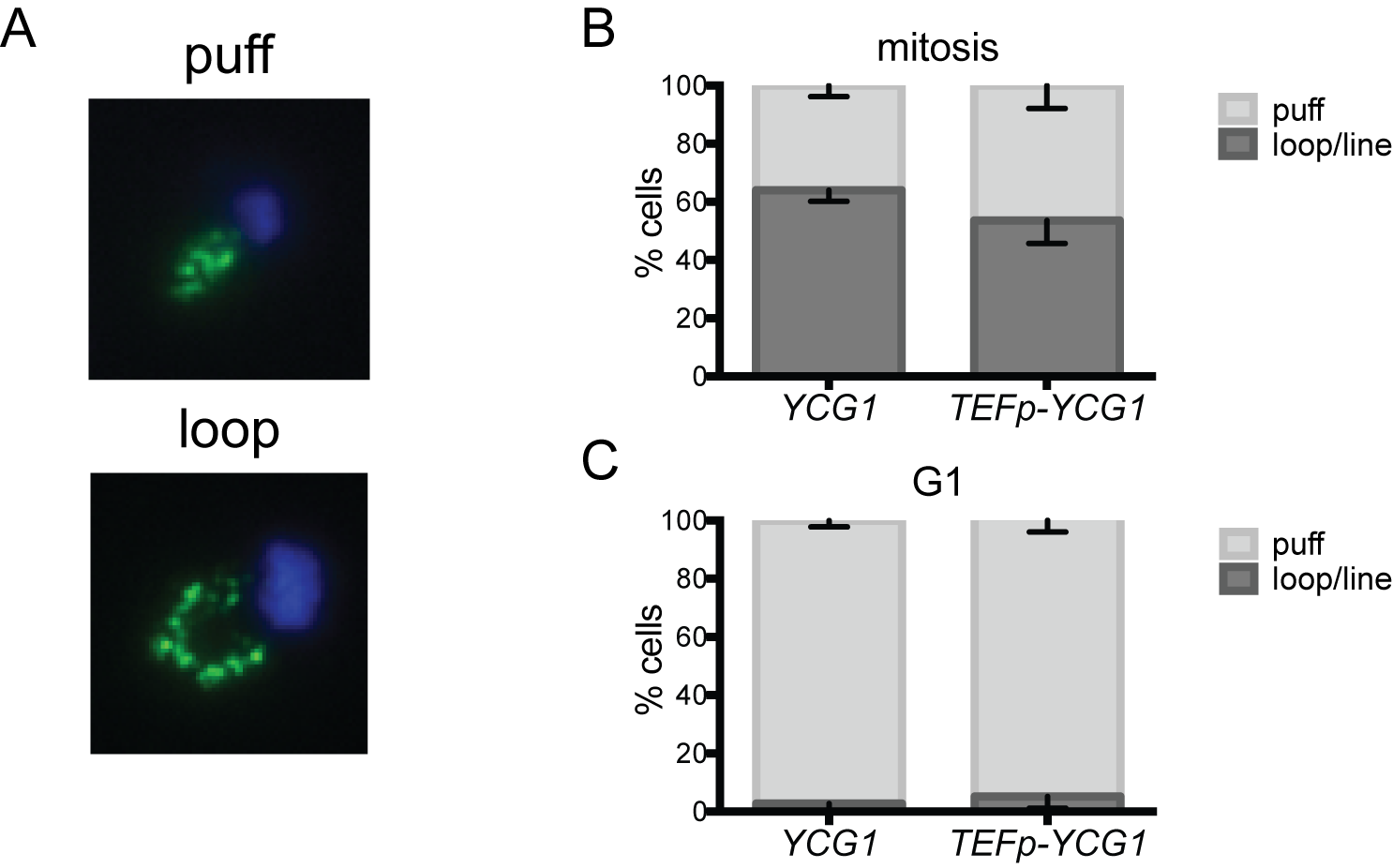

Supplement: S4 Fig — (A) Representative images of rDNA morphology as visualized by chromosome spreads. Cells were arrested in G1 by the addition of alpha-factor, or in metaphase by the addition of 20μg/ml nocodazole, for 3 hours. Spheroplasts were prepared and chromosomes spread on glass slides. Chromosomes were stained with DAPI and the rDNA was visualized by immunofluorescence to detect 3V5-tagged Net1, which is enriched on nucleolar DNA [22,47]. A puff represents decondensed DNA, whereas a loop represents condensed rDNA. (B) Percentages of rDNA puffs and loops/lines in wild-type (YJB653) and TEFp-YCG1 (YJB651) cells arrested in metaphase. In each experiment at least 130 cells were scored. Shown are the mean percentages +/- 1 standard deviation from n = 4 (YJB653) and n = 3 (YJB651) experiments. An unpaired t-test was used to confirm that there is no statistically significant difference between strains. (C) Percentages of rDNA puffs and loops/lines in wild-type (YJB653) and TEFp-YCG1 (YJB651) cells arrested in G1. In each experiment at least 100 cells were scored. Shown are the mean percentages +/- 1 standard deviation from n = 4 (YJB653) and n = 3 (YJB651) experiments. An unpaired t-test was used to confirm that there is no statistically significant difference between strains. (TIF) [file pgen.1006216.s004.tif]

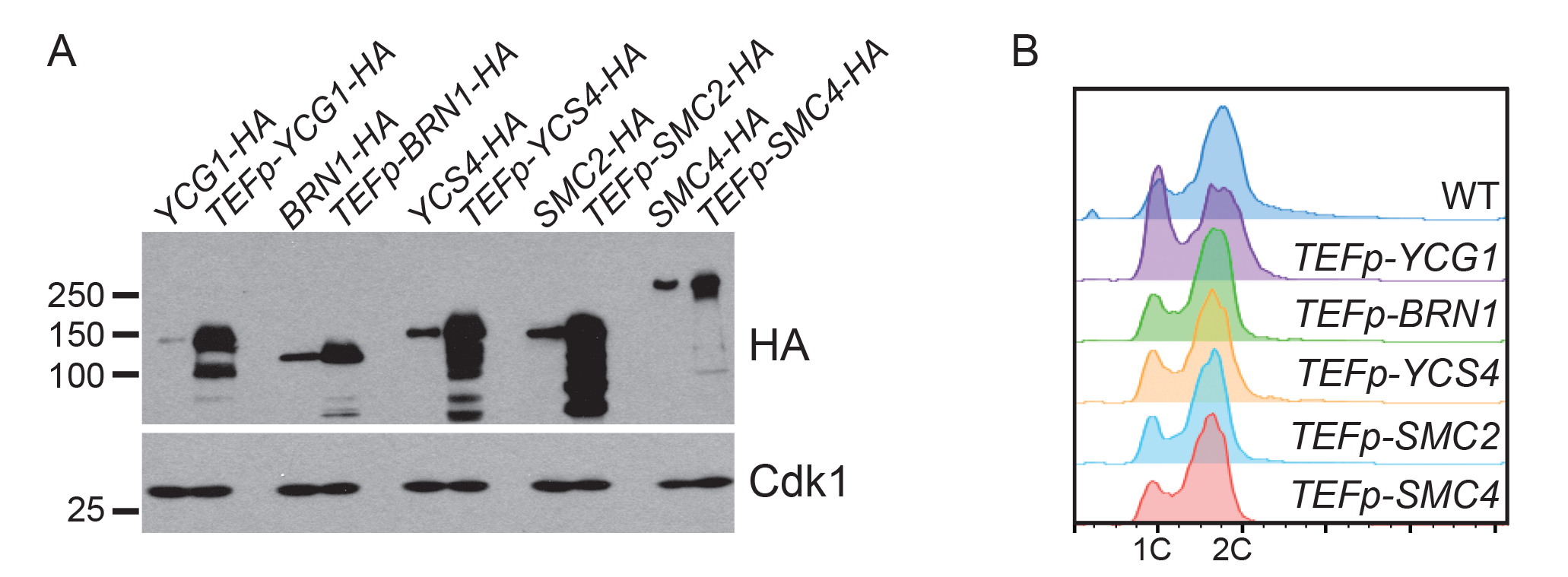

Supplement: S5 Fig — (A) Western blot showing relative expression of each 3HA-tagged condensin subunit in asynchronous wild-type (YTD33, YTD82, YTD83, YTD84, YTD80) and TEF1p knock-in (YTD336, YTD337, YTD353, YTD349, YTD362) strains. Cdk1 is shown as a loading control. (B) DNA content of asynchronous cultures as measured by flow cytometry showing the cell-cycle distributions of asynchronous cultures of the strains from (A). Note that overexpression of Ycg1, but not any other condensin subunit, results in a larger fraction of cells in G1 phase, consistent with a G1/S delay. (TIF) [file pgen.1006216.s005.tif]

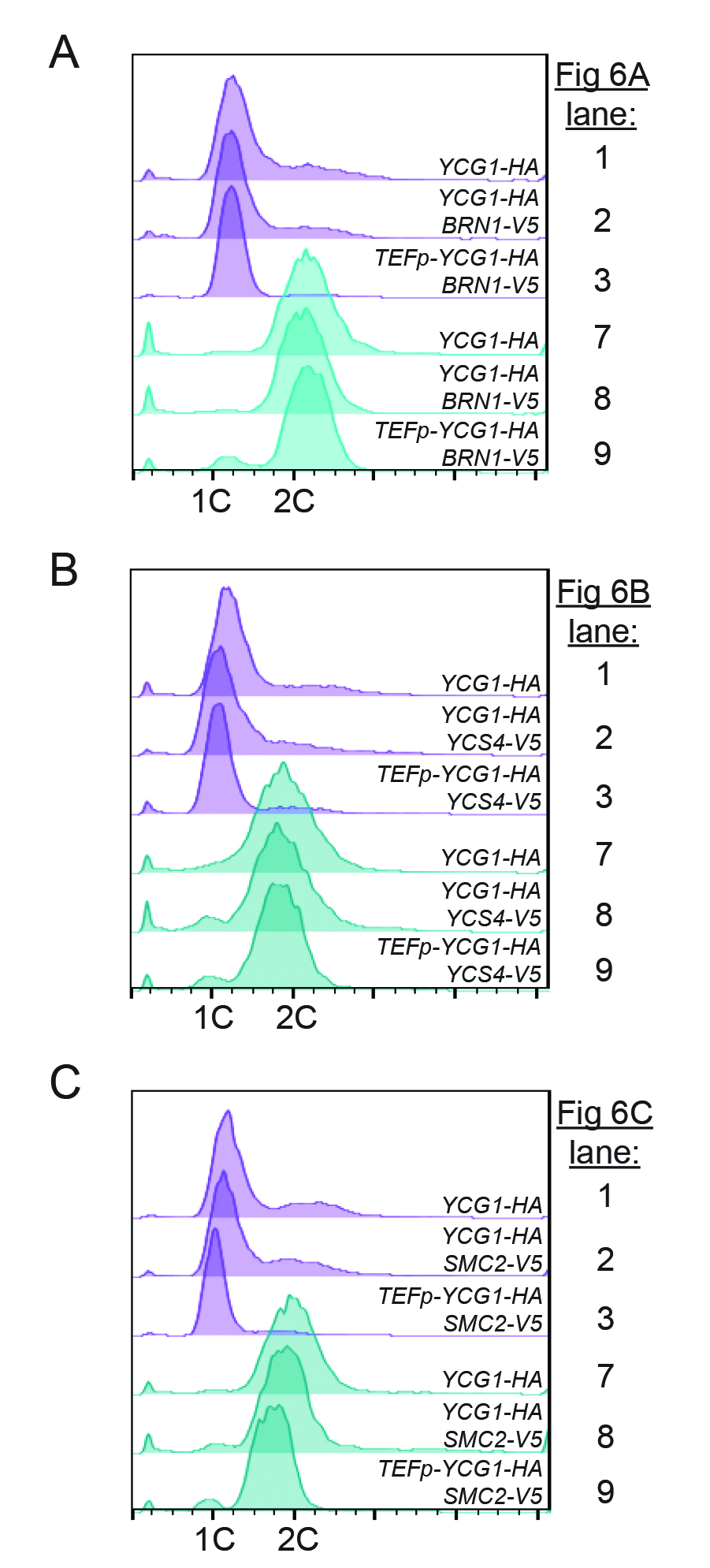

Supplement: S6 Fig — (A-C) G1 and mitotic arrests were confirmed by flow cytometry for the experiments shown in Fig 6A–6C, respectively. (TIF) [file pgen.1006216.s006.tif]

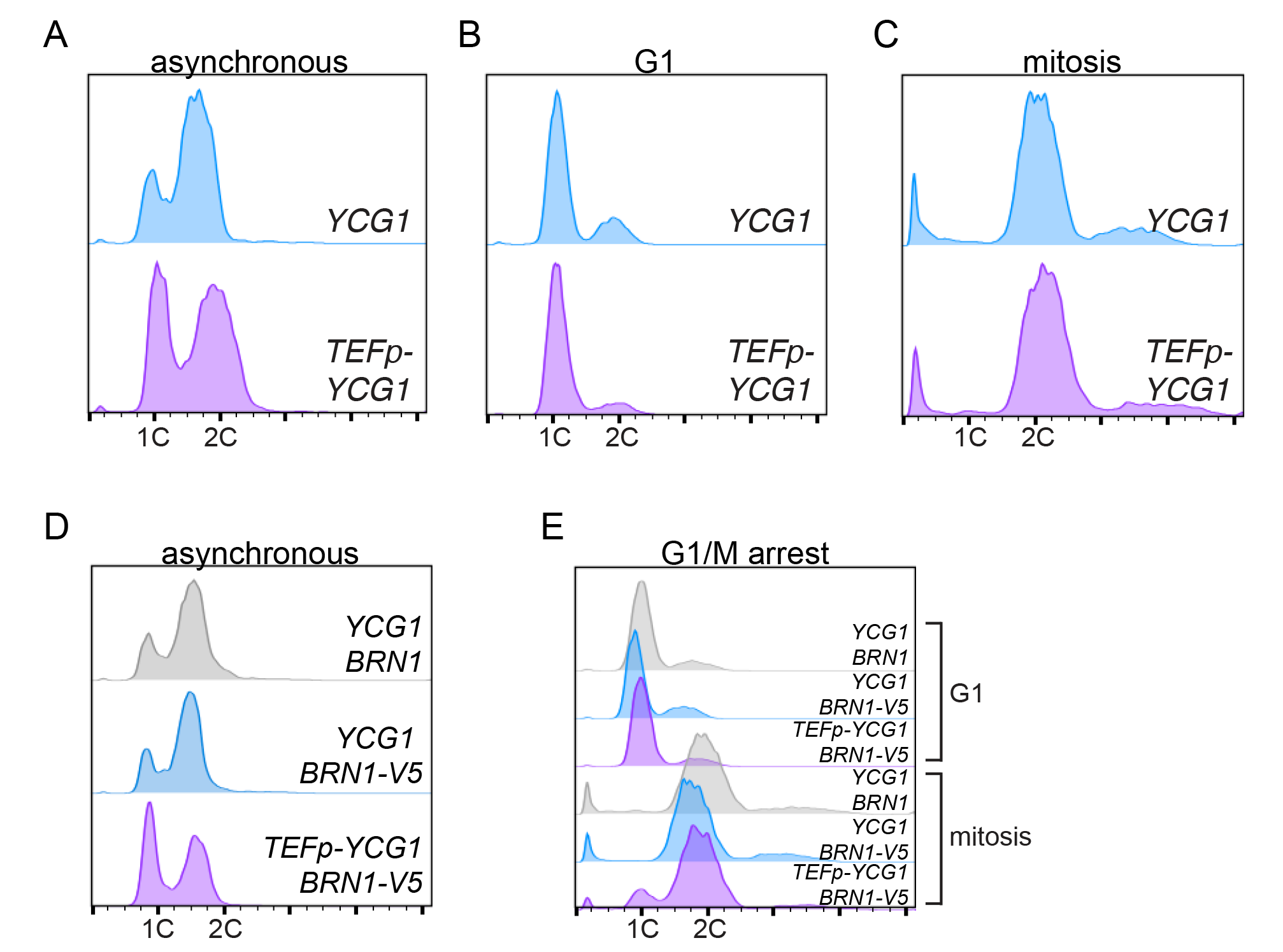

Supplement: S7 Fig — (A) DNA content analysis of asynchronous cells (YTD297, YTD342) from the chromosome spread assay shown in Fig 7. Note that there is a smaller fraction of cells in mitosis in the TEFp-YCG1 strain, so the increase in Ycg1 and Brn1 association with chromatin in this strain (Fig 7B and 7C) is not due to an increase in the number of mitotic cells. (B) DNA content analysis to confirm G1 arrest from a representative experiment included in Fig 7D. (C) DNA content analysis to confirm mitotic arrest from a representative experiment included in Fig 7E. (D) DNA content analysis to show cell cycle distributions from a representative experiment included in Fig 8A. (E) DNA content analysis to confirm G1 and mitotic arrest from a representative experiment included in Fig 8B. (TIF) [file pgen.1006216.s007.tif]
