# Supplementary material for: Levels of Ycg1 Limit Condensin Function during the Cell Cycle
Source: PLoS Genet. 2016 Jul 27;12(7):e1006216. doi: 10.1371/journal.pgen.1006216 (PMC4963108; doi:10.1371/journal.pgen.1006216)
Supplement: S1 Table — (PDF) [file pgen.1006216.s008.pdf]

**S1 Table. Strains list.**

| Name | Genotype | Background |
|---|---|---|
| YBL320 | *MATa his3Δ leu2Δ met15Δ ura3Δ YHP1-13Myc-LEU2 YOX1-3V5-KanMx TOS4-3FLAG-Hyg HCM1-3HA-HIS3* | S288C |
| YCG1-GFP | *MATa ura3Δ0 leu2Δ0 his3Δ1 met15Δ0 YCG1-GFP-HIS3* | S288C |
| YTD33 | *MATa ade2-1 his3-11,15 leu2-3,112 trp1-1 ura3-1 can1-100 YCG1-3HA-HIS3* | W303 |
| YTD82 | *MATa ade2-1 his3-11,15 leu2-3,112 trp1-1 ura3-1 can1-100 BRN1-3HA-HIS3* | W303 |
| YTD83 | *MATa ade2-1 his3-11,15 leu2-3,112 trp1-1 ura3-1 can1-100 YCS4-3HA-HIS3* | W303 |
| YTD84 | *MATa ade2-1 his3-11,15 leu2-3,112 trp1-1 ura3-1 can1-100 SMC2-3HA-HIS3* | W303 |
| YTD80 | *MATa ade2-1 his3-11,15 leu2-3,112 trp1-1 ura3-1 can1-100 SMC4-3HA-HIS3* | W303 |
| YUS5 | *MATa leu2-3, 112 ura3, his3-11,15 CanS GAL2 pup1-T30A pre3-T20A* | W303 |
| YTD43 | *MATa leu2-3, 112 ura3, his3-11,15 CanS GAL2 pup1-T30A pre3-T20A YCG1-3HA-HIS3* | W303 |
| YTD36 | *MATa ade2-1 can1-100 his3-11,15 leu2-3,112 trp1-1 ura3-1 ycg1Δ973-1035-3HA-HIS3* | W303 |
| YTD184 | *MATa ade2-1 his3-11,15 leu2-3,112 trp1-1 ura3-1 can1-100 ycg1Δ986-1035-3HA-HIS3* | W303 |
| YTD128 | *MATa ade2-1 his3-11,15 leu2-3,112 trp1-1 ura3-1 can1-100 ycg1Δ973-985-3HA-HIS3* | W303 |
| YTD148 | *MATa ade2-1 his3-11,15 leu2-3,112 trp1-1 ura3-1 can1-100 ycg1-K977A-3HA-HIS3* | W303 |
| YTD200 | *MATa ade2-1 his3-11,15 leu2-3,112 trp1-1 ura3-1 can1-100 ycg1-R976A-3HA-HIS3* | W303 |
| YTD201 | *MATa ade2-1 his3-11,15 leu2-3,112 trp1-1 ura3-1 can1-100 ycg1-R978A-3HA-HIS3* | W303 |
| YTD164 | *MATa ade2-1 his3-11,15 leu2-3,112 trp1-1 ura3-1 can1-100 ycg1-R980A-3HA-HIS3* | W303 |
| YTD336 | *MATa ade2-1 his3-11,15 leu2-3,112 trp1-1 ura3-1 can1-100 Hyg-TEF1p-YCG1-3HA-HIS3* | W303 |
| YTD337 | *MATa ade2-1 his3-11,15 leu2-3,112 trp1-1 ura3-1 can1-100 Hyg-TEF1p-BRN1-3HA-HIS3* | W303 |
| YTD353 | *MATa ade2-1 his3-11,15 leu2-3,112 trp1-1 ura3-1 can1-100 Hyg-TEF1p-YCS4-3HA-HIS3* | W303 |
| YTD349 | *MATa ade2-1 his3-11,15 leu2-3,112 trp1-1 ura3-1 can1-100 Hyg-TEF1p-SMC2-3HA-HIS3* | W303 |
| YTD362 | *MATa ade2-1 his3-11,15 leu2-3,112 trp1-1 ura3-1 can1-100 Hyg-TEF1p-SMC4-3HA-HIS3* | W303 |

| | | |
|---|---|---|
| YTD276 | *MATa ade2-1 his3-11,15 leu2-3,112 trp1-1 ura3-1 can1-100 YCG1-3HA-HIS3 NatMX6-MET3p-3HA-CDC20* | W303 |
| YTD361 | *MATa ade2-1 his3-11,15 leu2-3,112 trp1-1 ura3-1 can1-100 Hyg-TEF1p-YCG1-3HA-HIS3 NatMX6-MET3p-3HA-CDC20* | W303 |
| YTD290 | *MATa ade2-1 his3-11,15 leu2-3,112 trp1-1 ura3-1 can1-100 ycg1-K977A-3HA-HIS3 NatMX6-MET3p-3HA-CDC20* | W303 |
| YTD302 | *MATa ade2-1 his3-11,15 leu2-3,112 trp1-1 ura3-1 can1-100 Ycg1-3HA-HIS3 BRN1-3V5-kanMX6 NatMX6-MET3p-3HA-CDC20* | W303 |
| YTD355 | *MATa ade2-1 his3-11,15 leu2-3,112 trp1-1 ura3-1 can1-100 Hyg-TEF1p-YCG1-3HA-HIS3 BRN1-3V5-kanMX6 NatMX6-MET3p-3HA-CDC20* | W303 |
| YTD394 | *MATa ade2-1 his3-11,15 leu2-3,112 trp1-1 ura3-1 can1-100 Ycg1-3HA-HIS3 YCS4-3V5-kanMX6 NatMX6-MET3p-3HA-CDC20* | W303 |
| YTD395 | *MATa ade2-1 his3-11,15 leu2-3,112 trp1-1 ura3-1 can1-100 Hyg-TEF1p-YCG1-3HA-HIS3 YCS4-3V5-kanMX6 NatMX6-MET3p-3HA-CDC20* | W303 |
| YTD396 | *MATa ade2-1 his3-11,15 leu2-3,112 trp1-1 ura3-1 can1-100 Ycg1-3HA-HIS3 SMC2-3V5-kanMX6 NatMX6-MET3p-3HA-CDC20* | W303 |
| YTD397 | *MATa ade2-1 his3-11,15 leu2-3,112 trp1-1 ura3-1 can1-100 Hyg-TE1Fp-YCG1-3HA-HIS3 SMC2-3V5-kanMX6 NatMX6-MET3p-3HA-CDC20* | W303 |
| YTD297 | *MATa ade2-1 his3-11 leu2-3,112 trp1-1 ura3-1 can1-100 YCG1-3HA-HIS3 BRN1-3V5-KAN* | W303 |
| YTD342 | *MATa ade2-1 his3-11,15 leu2-3,112 trp1-1 ura3-1 can1-100 Hyg-TEFp-YCG1-HA-HIS3 BRN1-V5-KanMx6* | W303 |
| YTD199 | *MATa ade2-1 his3-11,15 leu2-3,112 trp1-1 ura3-1 can1-100 ycg1-S973A S975A T979A S981A-3HA-HIS3* | W303 |
| YTD176 | *MATa ade2-1 his3-11,15 leu2-3,112 trp1-1 ura3-1 can1-100 ycg1-E982A E984A-3HA-HIS3* | W303 |
| YTD284 | *MATa/MATα ade2/ade2 his3/his3 leu2/leu2 trp1/trp1 ura3/ura3 can1/can1 YCS4-13Myc-HIS3/YCS4* | W303 |
| YTD268 | *MATa/MATα ade2/ade2 his3/his3 leu2/leu2 trp1/trp1 ura3/ura3 can1/can1 YCG1-3V5-KanMX6/YCG1 YCS4-13Myc-HIS3/YCS4* | W303 |
| YTD269 | *MATa/MATα ade2/ade2 his3/his3 leu2/leu2 trp1/trp1 ura3/ura3 can1/can1 ycg1-K977A-3V5-KanMX6/YCG1 YCS4-13Myc-HIS3/YCS4* | W303 |
| YTD274 | *MATa/MATα ade2/ade2 his3/his3 leu2/leu2 trp1/trp1* | W303 |

| | | |
|---|---|---|
| | *ura3/ura3 can1/can1 SMC2-13Myc-HIS3/SMC2* | |
| YTD285 | *MATa/MATα ade2/ade2 his3/his3 leu2/leu2 trp1/trp1 ura3/ura3 can1/can1 YCG1-3V5-KanMX6/YCG1 SMC2-13Myc-HIS3/SMC2* | W303 |
| YTD267 | *MATa/MATα ade2/ade2 his3/his3 leu2/leu2 trp1/trp1 ura3/ura3 can1/can1 ycg1-K977A-3V5-KanMX6/YCG1 SMC2-13Myc-HIS3/SMC2* | W303 |
| YTD275 | *MATa/MATα ade2/ade2 his3/his3 leu2/leu2 trp1/trp1 ura3/ura3 can1/can1 SMC4-13Myc-HIS3/SMC4* | W303 |
| YTD286 | *MATa/MATα ade2/ade2 his3/his3 leu2/leu2 trp1/trp1 ura3/ura3 can1/can1 YCG1-3V5-KanMX6/YCG1 SMC4-13Myc-HIS3/SMC4* | W303 |
| YTD255 | *MATa/MATα ade2/ade2 his3/his3 leu2/leu2 trp1/trp1 ura3/ura3 can1/can1 ycg1-K977A-3V5-KanMX6 SMC4-13Myc-HIS3* | W303 |
| JS306 | *MATa his3Δ200 leu2Δ1 met15Δ0 trp1Δ63 ura3-167 RDN1::Ty1-MET15, mURA3/HIS3* | |
| JS576 | *MATa his3Δ200 leu2Δ1 met15Δ0 trp1Δ63 ura3-167 RDN1::Ty1-MET15, mURA3/HIS3 sir2Δ::KanMX4* | |
| YHA212 | *MATa his3Δ200 leu2Δ1 met15Δ0 trp1Δ63 ura3-167 RDN1::Ty1-MET15, mURA3/HIS3 YCG1-3HA-HIS3* | |
| YHA214 | *MATa his3Δ200 leu2Δ1 met15Δ0 trp1Δ63 ura3-167 RDN1::Ty1-MET15, mURA3/HIS3 ycg1-K977A-3HA-HIS3* | |
| YHA215 | *MATa his3Δ200 leu2Δ1 met15Δ0 trp1Δ63 ura3-167 RDN1::Ty1-MET15, mURA3/HIS3 Hyg-TEF1p-YCG1-3HA-HIS3* | |
| YTD430 | *MATa ade2-1 his3-11,15 leu2-3,112 trp1-1 ura3-1 can1-100 ycg1-K977A-3HA-HIS3 YCG1-3HA::URA3* | W303 |
| MW836a | *MATa ura3Δ0 leu2Δ0 his3Δ1 met15Δ0* | S288C |
| Y10100 | *MATa ura3Δ0 leu2Δ0 his3Δ1 met15Δ0 ycg1-2::KanMX6* | S288C |
| Y9804 | *MATa ura3Δ0 leu2Δ0 his3Δ1 met15Δ0 brn1-9::KanMX6* | S288C |
| YJB653 | *MATa ade2-1 his3-11 leu2-3,112 trp1-1 ura3-1 can1-100 YCG1-3HA-HIS3 NET1-3V5-KAN* | W303 |
| YJB651 | *MATa ade2-1 his3-11,15 leu2-3,112 trp1-1 ura3-1 can1-100 Hyg-TEFp-YCG1-HA-HIS3 NET1-V5-KanMx6* | W303 |
