# Supplementary material for: Levels of Ycg1 Limit Condensin Function during the Cell Cycle
Source: PLoS Genet. 2016 Jul 27;12(7):e1006216. doi: 10.1371/journal.pgen.1006216 (PMC4963108; doi:10.1371/journal.pgen.1006216)
Supplement: S2 Table — (PDF) [file pgen.1006216.s009.pdf]

S2 Table. qPCR primer list.

Primer	Location	Sequence	Reference
CEN5DZF	Condensin-depleted region in *SEC3* on Chr V	TTTCCTCTCCTCCTGCTTTATTC	this study
CEN5DZR	Condensin-depleted region in *SEC3* on Chr V	GAGGCTGAACTGAGACGATTAG	this study
rDNA9-F	rDNA, intergenic spacer 1	AGCCTACTCGAATTCGTTTCC	Clemente-Blanco et al, *Nat Cell Biol*, 2011
rDNA9-R	rDNA, intergenic spacer 1	ATAGTGAGGAACTGGGTTACC	Clemente-Blanco et al, *Nat Cell Biol*, 2011
CEN4-F	Chr IV centromere	CCGAGGCTTTCATAGCTTA	Bizzari and Marston, *J Cell Biol*, 2011
CEN4-R	Chr IV centromere	ACCGGAAGGAAGAATAAGAA	Bizzari and Marston, *J Cell Biol*, 2011
CEN12-F	Chr XII centromere	CTTTACGCGGGTGTGTACTT	this study
CEN12-R	Chr XII centromere	CAACCAAACTGGTGTATGCTAATATC	this study
Tel0.6F	Chr VI telomere, right arm	CAGGCAGTCCTTTCTATTTC	Hoppe et al, *Mol Cell Biol*, 2002
Tel0.6R	Chr VI telomere, right arm	GCTTGTTAACTCTCCGACAG	Hoppe et al, *Mol Cell Biol*, 2002
